# Promoting •OH-dominant Fenton-like process over peracetic acid activation by ultrafine FeO_x_ nanoclusters anchored carbonaceous nanosheets

**DOI:** 10.1016/j.fmre.2023.05.007

**Published:** 2023-05-30

**Authors:** Qian Hu, Taoyu Yang, Shanli Wang, Licong Xu, Minghua Wu, Deyou Yu, Kaixing Fu, Jinming Luo

**Affiliations:** a Engineering Research Center for Eco-Dyeing and Finishing of Textiles (Ministry of Education), College of Textile Science and Engineering, Zhejiang Sci-Tech University, Hangzhou 310018, China; b School of Environmental Science and Engineering, Shanghai Jiao Tong University, Shanghai 200240, China; c Zheijiang Sci-Tech University Xiangshan Research Institute, Xiangshan 315700, China

**Keywords:** Peracetic acid, Fenton-like, AOPs, Nanocluster, Selectivity

## Abstract

Peracetic acid (PAA) has recently been considered a promising oxidant candidate for heterogeneous Fenton-like reactions; however, the main generation and contribution of organic radicals (R-O•) with unsatisfactory oxidation potential compromises wastewater decontamination efficiency. In this study, we demonstrate the rational design and synthesis of ultrafine FeO_x_ nanocluster-anchored carbonaceous nanosheets (UFe-CN) for altering the PAA activation pathway from R-O• to •OH dominant process via *in situ* framework collapse carbonization of MIL-53(Fe). The constructed UFe-CN/PAA system effectively accelerated refractory micropollutant (*e.g.*, p-nitrophenol (4-NP)) decomposition by the enhanced •OH formation (up to 65.24 µmol *L*^−1^) under a wide pH range (3.0–9.0), outperforming the benchmark iron-based catalyst counterparts by 4.2–10.8 times. This outstanding Fenton-like catalytic activity of UFe-CN is primarily attributed to the significant improvement in electron mitigation, *ca.* 49 times higher than that of its MIL-53(Fe) counterpart, for interface catalysis reactions triggered by iron species cycling. Furthermore, to facilitate adaptive engineering, the organic pollutant removal efficiency could be easily tuned by varying several key treatment factors, including the initial pH, PAA concentration, and UFe-CN dosage. More importantly, the excellent practicality of UFe-CN/PAA was demonstrated by systematically evaluating the impact of the water matrix, catalyst regeneration capability, and wastewater treatment efficiency. Overall, this study provides a significant understanding of •OH-dominated PAA activation and an effective catalyst development paradigm to facilitate the practical application of PAA-based Fenton-like oxidation.

## Graphical abstract

**Figure:**
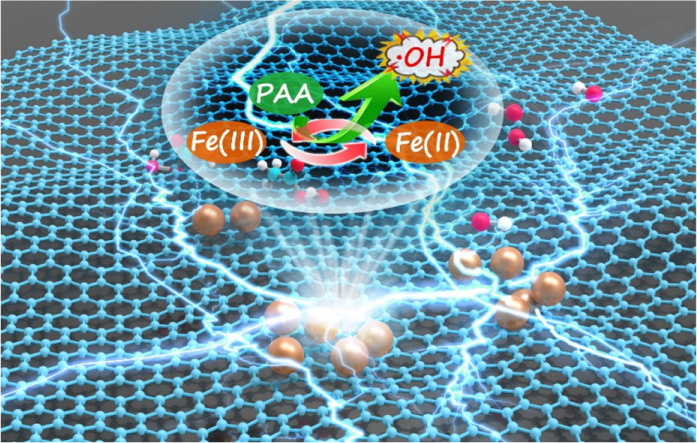

## 1 Introduction

Phenolic compounds represent an important and essential class of intermediate products in modern industrial applications such as drugs, insecticides, dye manufacturing, and textile and tannery coloring [1,2]. Owing to the rapidly increasing demand for these activities, phenolic micropollutants (*e.g.*, p-nitrophenol (4-NP)) with high persistence, bioaccumulation, and toxicity to humans and animals have been widely observed in industrial/domestic wastewater and natural water, thus receiving extensive attention regarding their effective removal [3], [4], [5]. As a more practicable and feasible technique for refractory contaminant elimination than other methods (*e.g.*, adsorption and membrane separation), advanced oxidation processes (AOPs) are capable of generating highly reactive oxygen species (ROS) to degrade micropollutants [6], [7], [8], [9], [10], [11]. In particular, Fenton-like processes triggered by the heterolytic and homolytic cleavage of peroxides have shown greater potential in the broadband abatement of recalcitrant pollutants [12].

The exploration of suitable oxidants for Fenton-like processes has recently attracted wide interest, especially because of the compromising costs and compatible facilities [13]. Peroxide-relevant oxidants, including hydrogen peroxide (H_2_O_2_) [14], [15], [16], [17], peroxymonosulfate (PMS) [18], [19], [20], peroxydisulfate (PDS) [21], [22], [23], and sodium percarbonate (SPC) [24], [25], [26] are important suppliers of ROS through diverse activation methods (*e.g.*, light, heating, and/or catalyst incorporation). Nevertheless, the application and activation of these peroxides in Fenton-like processes continue to encounter several critical issues, such as high energy input, extensive chemical consumption, and potential secondary pollution [12,19,27,28]. Consequently, peracetic acid (CH_3_C(O)OOH, PAA), a typical and powerful disinfection oxidant [9], is emerging as a promising source for delivering radicals because of its lower activation energy for dissociating the O—O bond (159 kJ mol^−1^) and lower risk of forming harmful byproducts [13]. Unfortunately, PAA demonstrates specific selectivity in the decomposition of numerous refractory pollutants because of its moderate redox potential (E ^0^_PAA_ = 1.06–1.96 V). Therefore, significant efforts have been made to activate PAA to generate organic (R-O•) and hydroxyl radicals (•OH).

Generally, PAA can be easily activated by external energy (*e.g.*, light [29,30], heating [31], ultrasound [32], and microwaves [33]) or transition metal ions (*e.g.*, Fe^2+^ [34], Co^2+^ [35], and Ru^3+^ [36]), which have been identified as homogeneous methods for inducing multiple radical generation. As an illustration, the O—O bond in the PAA molecule is deemed to undergo homolysis splitting under UV irradiation [29], simultaneously forming R-O• and •OH (Eq. 1) responsible for removing micropollutants. However, transition metal ions allow the catalytic decomposition of PAA into R-O• (*e.g.*, CH_3_C(O)O• and CH_3_C(O)OO•) through a one-electron transfer pathway (Eqs. 2 and 3) with little or no •OH production [8]. R-O•, which has a longer half-life and higher tolerance to co-existing matrices than •OH, normally fails to destroy refractory phenolic compounds featuring electron deficiency at aromatic nuclei owing to its relatively low oxidation capacity. The homogeneous activation systems established by the above methods are largely limited by three critical issues: (1) compatible facilities, (2) aqueous metal ion disposal, and (3) catalyst regeneration [8,13]. One of the most promising strategies to overcome these drawbacks is to rationally design and develop heterogeneous catalysts instead of using an external energy input or metal ion addition.

(1) $${\mathrm{CH}}_{\text{3}}{\mathrm{CO}}_{3}H+\mathrm{UV}\to {\mathrm{CH}}_{3}{\mathrm{CO}}_{2}•\;+\;•\mathrm{OH}$$

(2) $${\mathrm{CH}}_{3}{\mathrm{CO}}_{3}H+M^{n+}\to {\mathrm{CH}}_{3}{\mathrm{CO}}_{2}•\;+\;{\mathrm{OH}}^{-}+M^{\left(n+1\right)+}$$

(3) $${\mathrm{CH}}_{3}{\mathrm{CO}}_{3}H+M^{\left(n+1\right)+}\to {\mathrm{CH}}_{3}{\mathrm{CO}}_{3}•\;+\;H^{+}+M^{n+}$$

Recently, metal-free carbonaceous materials (*e.g.*, biochar, activated carbon, carbon fibers, and reduced graphene oxide) [37], [38], [39], [40] and metal-based candidates (*e.g.*, metal oxides, molybdenum disulfide, Co@MXenes, ZIF-67, and single-atom-doped C_3_N_4_) [41], [42], [43], [44], [45] have been proven to activate PAA effectively. However, the development of highly efficient catalysts for PAA activation remains a challenge. The diverse performances and pathways toward PAA activation in metal-free and metal-based catalysts indicate potential improvements. Specifically, the unique microstructure of the highly conductive carbon rings within the metal-free catalysts favors electron-transfer-dominated interfacial catalytic reactions during PAA activation. Nevertheless, the unsustainable supply of infinite and unrecyclable electron-donating groups (*e.g.*, C=O and C—OH) with weak activity mitigates the overall efficiency and practicality. In contrast, the acceleration of PAA activation using metal-based catalysts depends on the density of the highly active metal sites, which is negatively influenced by the aggregation of metal-based nano/microparticles. Inspired by these findings, the synergistic coupling of the advantages of highly-conductive microstructure with confined active metal sites to develop a class of “two-in-one” heterogeneous catalysts would be an appealing paradigm for efficient PAA activation. More importantly, this is a significant field that has rarely been explored or advanced for water decontamination using PAA-based AOPs.

In this study, we proposed a novel and efficient heterogeneous PAA activation system for the degradation of refractory phenolic compounds by incorporating a rationally designed ultrafine FeO_x_ nanocluster-anchored carbonaceous nanosheets (UFe-CN). Specifically, the former component was spatially separated to fully expose the active iron sites, while the latter served as a highly conductive electron-transfer network to facilitate the interfacial catalytic reaction. UFe-CN was constructed by an *in situ* framework collapse carbonization method using MIL-53(Fe) as the precursor because of its intriguing features for delivering carbonaceous derivatives with well-maintained nanoporous texture and isolated FeO_x_ nanoclusters. Unlike previously reported heterogeneous catalyst analogies, the UFe-CN was found to alter the PAA activation pathway from R-O• to •OH dominant process, mainly responsible for 4-NP degradation under a wide pH range (3.0–11.0), outperforming the benchmark iron-based catalyst counterparts by 4.2–10.8 times. In addition, the key environmental parameters that potentially affected the degradation efficiency of the UFe-CN/PAA system were systematically investigated and elucidated. The practicality of the established UFe-CN/PAA system was further demonstrated by evaluating its effects on real water matrices, catalyst regeneration capability, and wastewater treatment efficiency. Our study provides a novel protocol for constructing UFe-CN that can effectively activate PAA and provides significant insights into the promotion of the •OH-dominant degradation process controlled by a rapid electron-transfer-assisted interface catalysis reaction.

## 2 Materials and methods

### 2.1 Chemicals

Peracetic acid (PAA, *ca*. 15%), p-phthalic acid (H_2_BDC, C_8_H_6_O_4_), iron chloride hexahydrate (FeCl_3_·6H_2_O), p-benzoquinone (BQ, C_6_H_4_O_2_), 4-amino-2,2,6,6-tetramethylpiperidine (TEMP, C_9_H_20_N_2_), 5,5-dimethyl-1-pyrroline N-oxide (DMPO, C_6_H_11_NO), coumarin (COU, C_9_H_6_O_2_), methanol (MeOH, CH_3_OH), and humic acid (HA, ≥ 90%) were purchased from Aladdin Chemical Co., Ltd. (Shanghai, China). N, N-dimethylformamide (DMF, C_3_H_7_NO), ethanol (C_2_H_6_O), chlorohydric acid (HCl, 37.5wt%), sodium hydroxide (NaOH), sodium carbonate (Na_2_CO_3_), and sodium chloride (NaCl) were purchased from Hangzhou Gaojing Chemical Co., Ltd. (Hangzhou, China). Tert‑butyl alcohol TBA (C_4_H_10_O) was obtained from Shanghai Macklin Biochemical Co., Ltd. (Shanghai, China). All chemicals were of analytical grade and were used without further purification. The ultrapure water (UW) used in the experiment was produced using a Milli-Q Advantage A10 (Millipore). In addition, two real wastewater samples (denoted as A and B) were collected from Zhejiang Daneng Textile Dyeing Co., Ltd. (China) to evaluate the practicality of the constructed heterogeneous PAA activation system.

### 2.2 Fabrication of UFe-CN

UFe-CN was prepared via an *in situ* framework collapse carbonization method using MIL-53(Fe) as the precursor. MIL-53(Fe) was synthesized using a solvothermal process according to our previous study without any modifications [46]. Subsequently, the received MIL-53(Fe) was subjected to annealing at 500 °C for 3 h under N_2_ protection, which allows for the in-situ collapse of Fe(III)-BDC framework since its thermal decomposition temperature was approximately 350 °C. The formed MOF-derived iron species/carbon composite powder was thoroughly washed several times using UW to eliminate the residual free iron ions. Finally, the washed black powder sample was separated by centrifugation (10,000 rpm, 5 min) and vacuum-dried at 100 °C for 12 h to afford the final UFe-CN product. Benchmark iron species commercially obtained from Sigma Aldrich Co., Ltd., such as zero-iron powder, α-Fe_2_O_3_, and Fe_3_O_4,_ were also used for comparison.

### 2.3 Characterization of UFe-CN

The microstructure of UFe-CN was comprehensively revealed using a series of advanced characterization methods, including field emission scanning electron microscopy (FESEM), transmission electron microscopy (TEM), nitrogen gas adsorption-desorption measurements, powder X-ray diffraction (PXRD), Fourier transform infrared (FT-IR) spectroscopy, X-ray photoelectron spectroscopy (XPS), and electrochemical analyses. Field emission scanning electron microscopy (FESEM) and transmission electron microscopy (TEM) images of the prepared UFe-CN were obtained using a Zeiss Ultra55 (Germany) scanning electron microscope and an FEI Tecnai F20 transmission electron microscope (USA), respectively. The PXRD patterns of prepared UFe-CN and other benchmark counterparts were recorded on a Thermo ARL-XTRA (USA) diffractometer over at a 2θ range of 5°–80°. A Bruker Vertex 70 (Germany) spectrometer was applied to collect the FT-IR spectra of these catalysts, while the XPS spectra were obtained on a Thermo Fisher Scientific K-Alpha (USA) instrument to elucidate the chemical composition and pivotal iron atom speciation. Nitrogen gas adsorption-desorption measurements were performed using a Micromeritics ASAP 2020 (USA) gas adsorption system to obtain the sorption-desorption isotherms. Linear sweep voltammetry (LSV) plots, electrochemical impedance spectra (EIS), and Tafel polarization curves of the prepared catalysts were obtained using a CHI660 electrochemical workstation (China).

### 2.4 Heterogeneous fenton-like catalysis

The catalytic performance of the prepared UFe-CN was compared with that of MIL-53(Fe) and other benchmarks using refractory 4-NP as the target compound. All the Fenton-like reactions were conducted in a glass reactor (100 mL) with constant stirring (*ca.* 200 rpm) at a temperature of 20 °C in the dark condition to avoid the effect of photo-induced decomposition of PAA. The initial pH value (pH_0_) of the solutions was adjusted to 7.0 using 0.1 M HCl or 0.1 M NaOH aqueous solutions unless otherwise stated. Typically, 0.02 g of catalyst was added to a 4-NP solution (50 mL, 20 mg *L*^−^^1^), followed by the addition of a certain amount of PAA to initiate the heterogeneous Fenton-like process. Subsequently, 2.0 mL aliquots of reaction suspension were withdrawn and filtered using a PTFE syringe filter at a constant time interval before adding 0.5 mmol TBA (equal to 0.25 M) to quench the PAA or other ROS immediately. The key environmental parameters potentially affecting or manipulating the overall decontamination efficiency of the UFe-CN/PAA activation system were systematically investigated by alerting the single variable, such as pH_0_ (3.0–11.0), PAA dosage (0.10–1.00 mM), and UFe-CN concentration (0.1–0.6 g *L*^− 1^). In addition, certain amounts of widely found water matrices, including CO_3_^2−^, Cl^−^, and HA, were individually added to a typical catalytic runner to study their possible effects on the UFe-CN/PAA activation system. For the recycling experiment, the spent UFe-CN was collected and recovered by filtration, with washing with UW and ethanol several times before being dried overnight at 100 °C. Each heterogeneous Fenton-like process was performed in triplicates.

### 2.5 Analytical methods

The 4-NP concentration was monitored using an Agilent Technologies Series 1260 high-performance liquid chromatograph (HPLC, USA) equipped with a UV–vis detector. The effective wavenumber was set at 320 nm, and a ZORBAX Eclipse XDB-C18 column (3.5 µm, 4.6 mm × 150 mm) was employed as the stationary phase. A mobile phase formula of 30% 2 mM H_3_PO_4_ aqueous solution and 70% pure MeOH was applied with a flow rate of 1.00 mL min^−^^1^. The concentration of the leached iron ions was determined using an Agilent 720 mass spectrometer equipped with inductively coupled plasma mass spectrometry (ICP-MS, USA). Quenching experiments and electron paramagnetic resonance (EPR) measurements were conducted to analyze the species and contributions of ROS to the UFe-CN/PAA system. In the case of the quenching experiments, TBA, an effective •OH scavenger, was employed to distinguish the role of •OH, whereas MeOH was used to identify the function of surface-bound or free •OH. Electron paramagnetic resonance (EPR) spectra were recorded on a Bruker A300 spectrometer (Germany) using DMPO as the probe for •OH and R-O• at room temperature. The accumulated concentration of •OH was determined using a fluorescence method with coumarin as the probe; detailed information is presented in Text S1 (Supporting Information). A Hitachi F-4600 fluorescence spectrophotometer (Japan) was employed to reveal the three-dimensional fluorescence excitation-emission matrix spectrum (3D EEMs) of the real wastewater samples before and after treatment with the UFe-CN/PAA heterogeneous Fenton-like process.

## 3 Results and discussion

### 3.1 Fabrication and characterization of UFe-CN

**Fig. 1:**
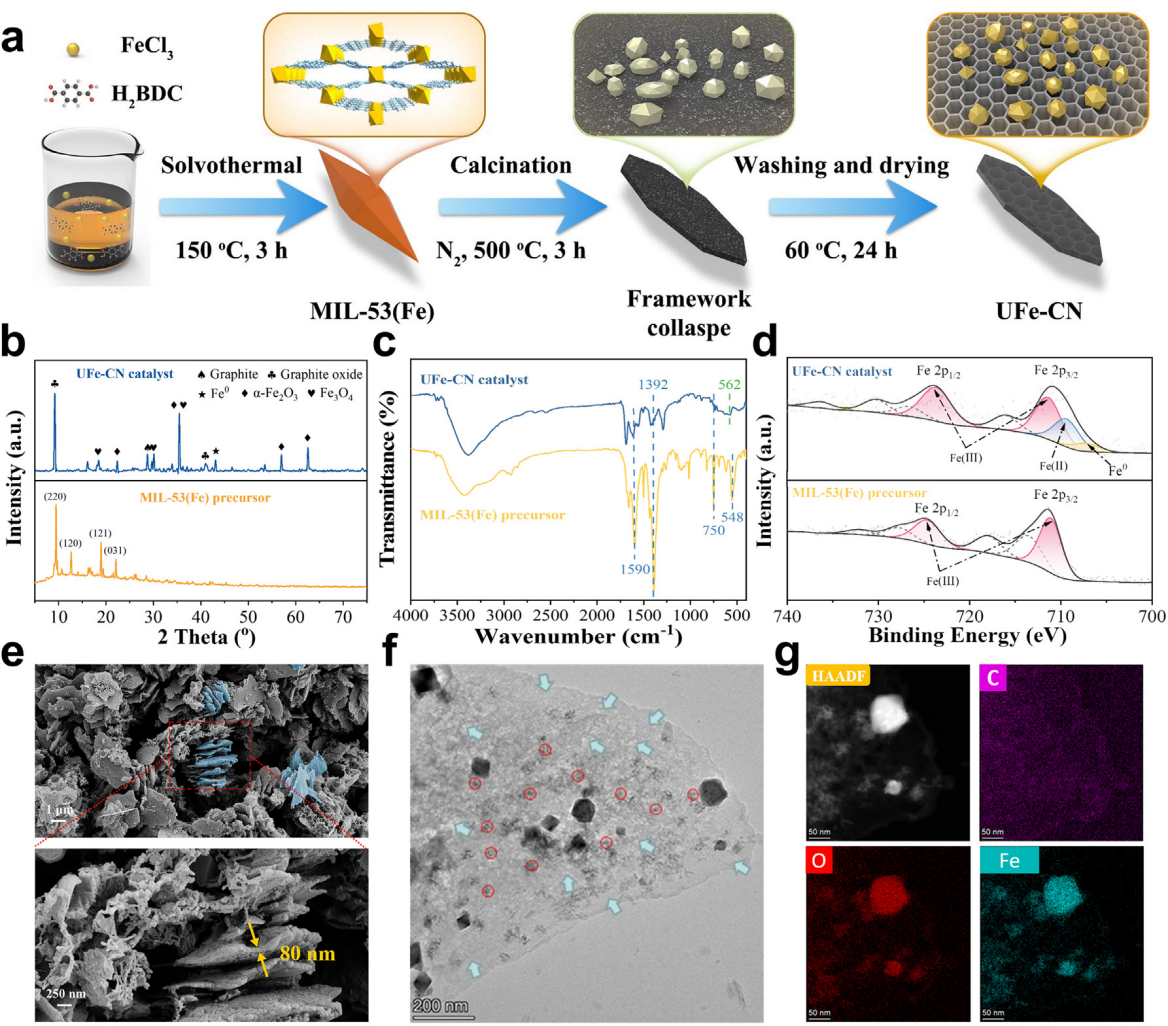
**Preparation and physicochemical structure illustrations of the UFe-CN catalyst.** (a) Schematic of in-situ framework collapse carbonization method for UFe-CN catalyst preparation. (b) PXRD patterns, (c) FT-IR spectra, and high-resolution Fe 2p XPS spectra of the UFe-CN catalyst and MIL-53(Fe) precursor. Four characteristic peaks observed in PXRD pattern of MIL-53(Fe) precursor refer to its (220), (120), (121), and (031) facets. (e) Nanosheet morphology of UFe-CN catalyst unraveled by FESEM images with lower (up) and higher (down) magnifications. (f) TEM image of UFe-CN catalyst that further confirms the nanosheet construction. Red circles and blue arrows indicate the FeO_x_ nanoclusters and nanoporous channels. (g) HAADF-STEM and EDS mapping images of FeO_x_ nanoclusters anchored in a MOF-derived porous carbon matrix. The bright dots represent for FeO_x_ nanoclusters, whose particle size distribution is illustrated in Fig. S10. Corresponding EDS mapping of C, O, and Fe elements revealing the homogeneous distribution of FeO_x_ nanoclusters on the carbon support.

The specific protocol for the *in situ* framework collapse carbonization method for UFe-CN is schematically illustrated in Fig. 1a. Owing to the outstanding advantages of MIL-53(Fe) microstructures, the MIL-53(Fe) precursor was first synthesized via a solvothermal approach to provide an intrinsic porous organic-inorganic framework and well-isolated iron-oxo clusters for delivering UFe-CN rationally. Afterward, the thermal actuation pyrolysis with the assistance of an inert N_2_ atmosphere at 500 °C allowed the collapse carbonization of the porous framework owing to the self-depletion of intramolecular or intermolecular C, O, and H elements. Meanwhile, the well-isolated iron-oxo clusters experienced thermodynamic aggregation without the support of organic linkers and partial reduction in the presence of a carbon substrate.

The PXRD pattern of the prepared MIL-53(Fe) (Fig. 1b) matched well with previous studies regarding the 2θ locations of three distinct peaks (*i.e.*, 9.4°, 17.6°, and 18.6°), suggesting the successful synthesis of functional MIL-53(Fe) [46]. Considering the in-situ framework collapse carbonization process, significant changes in the crystalline phase of MIL-53(Fe) were observed in the PXRD pattern of UFe-CN (Fig. 1b). Specifically, UFe-CN contained various iron species (FeO_x_), including Fe^0^ (JCPDS-00–006–0696), α-Fe_2_O_3_ (JCPDS-00–024–0072), and Fe_3_O_4_ (JCPDS-01–089–0950), according to the characteristic diffraction peaks marked with geometric shapes. Notably, in Fig. 1b, the two peaks at 9.1° and 28.4° are assigned to the (001) plane of graphite oxide and the (002) plane of the hexagonal crystal structure of expanded graphite, respectively. This result demonstrates the inherently highly conductive microstructure architecture for rapid electron transfer [47,48]. Compared to pure graphite oxide (2θ = *ca*. 11.0°) (XRD of pure graphite oxide), the corresponding peak in Fig. 1b showed a slight shift (Δ_2θ_ = 1.9°) to a lower 2θ position, which was likely caused by the presence of strong metal-support interaction.

To uncover the in-depth behavior of organic linkers and iron-oxo clusters, we employed FT-IR to detect changes in the chemical structures of MIL-53(Fe) before and after the *in situ* framework collapse carbonization process. As shown in Fig. 1c, a fingerprint region [49] for the symmetric and asymmetric vibrations of the C=O groups was recognized at 1392 and 1590 cm^−^^1^ in the FT-IR spectra of UFe-CN and MIL-53(Fe) with different peak intensities. The confirmed C=O groups indicated the partial oxidation of the graphite substrate in UFe-CN, whereas the evidently weakened intensity revealed a greater loss of C=O groups, as it is likely a source for H_2_O or CO_2_ gas formation during carbonization. In addition, the bending vibration of the C—H groups (750 cm^−^^1^) in the benzene ring of MIL-53(Fe) completely disappeared after carbonization. This indicates that the benzene ring underwent an H-extraction step to form graphite with partially oxidized hexagonal carbon arrays. However, the rational conversion of Fe-O clusters to other FeO_x_ forms was identified by the disappearance of the characteristic signal (548 cm^−^^1^) in the spectrum of UFe-CN and the generation of a new weak peak at approximately 562 cm^−^^1^.

We further explored the elemental composition and chemical state of the prepared UFe-CN using X-ray photoelectron spectroscopy (XPS). The XPS survey profiles in Fig. S1 indicate that UFe-CN and MIL-53(Fe) mainly comprise C, O, and Fe. The atomic content of the C element had a large increase after the carbonization, supporting our deduction of the growth of graphite oxide substrate. The C 1s spectrum (Fig. S2) of UFe-CN was similar to the counterpart, showing two identical peaks for the sp^2^-hybridized carbon and C=O group at 284.8 and 288.8 eV, respectively [50]. UFe-CN and MIL-53(Fe) also shared a consistent O 1s spectrum (Fig. S3), where a prominent peak at 531.9 eV was observed and attributed to the overlap of the O-Fe and O=C groups. As shown in Fig. 1d, the high-resolution Fe 2p XPS spectrum of MIL-53(Fe) contains two typical peaks of Fe 2p_3/2_ and Fe 2p_1/2_ at binding energies (BE) of 711.1 and 725.0 eV, respectively [51,52]. In addition, the observed two peaks at 718.0 and 731.6 eV could be attributed to the shakeup satellite of Fe(III) [53]. These results suggest that the Fe in the Fe-O cluster of MIL-53(Fe) is predominantly present in a trivalent state. Nevertheless, besides the similar fingerprint peaks of Fe(III) recognized in MIL-53(Fe), two new peaks at 709.6 and 707.3 eV were evidently observed in the Fe 2p XPS spectrum of UFe-CN, corresponding to Fe(II) and Fe^0^, respectively. By merging the PXRD and FT-IR analyses, the Fe(III) center in MIL-53(Fe) was subjected to partial reduction after framework collapse carbonization to afford hybrid iron species such as Fe^0^, Fe_3_O_4_, and α-Fe_2_O_3_, which may favor the enduring and rapid cycle of the Fe(III)/Fe(II) redox couples.

Field-emission scanning electron microscopy (FESEM) and transmission electron microscopy (TEM) were used to reveal the morphology of the prepared catalysts. The FESEM images (Fig. 1e) demonstrate that UFe-CN has a uniform carbonaceous nanosheet (*ca.* 80 nm thick) morphology with rough surfaces probably decorated by FeO_x_ nanoclusters (Fig. S4). However, MIL-53(Fe) is characterized by a smooth, regular, dodecahedral crystalline shape with a length and width of 1 µm × 2 µm (Fig. S5). Such unique microstructures of UFe-CN, derived from the collapsed carbonization of the MIL-53(Fe) framework, can greatly favor PAA activation because it is more accessible to iron sites. Numerous FeO_x_ nanoclusters in the relatively dark regions marked with red circles, as well as distinctive nanoporous channels in the relatively bright regions marked by blue arrows, can be easily observed in the TEM images (Figs. 1f and S6) of UFe-CN. Characteristic d-spacing values of 2.59 and 2.42 Å found in FeO_x_ nanoclusters (Figs. S7 and S8), which were consistent with the theoretical radius of α-Fe_2_O_3_ (110) planes (2.52 Å, JCPDS-00–024–0072) and Fe_3_O_4_ (222) planes (2.42 Å, JCPDS-01–089–0950), clarified the hybrid composition of FeO_x_ nanoclusters as PXRD analyzed. It is difficult to measure d-spacing values identical to the various theoretical radii of the Fe^0^ facets (JCPDS-00–006–0696) owing to the margin ratio of Fe^0^. Notably, as depicted in Figs. 1f and S9, the spatial separation from the integrated MIL-53(Fe) is considered a reliable evolution step to generate FeO_x_ nanoclusters during carbonization. In addition, the atomic structure of the FeO_x_ nanoclusters anchored in the carbonaceous nanosheet derived from the iron-oxo nodes in MIL-53(Fe) was further examined using high-angle annular dark-field (HAADF) scanning transmission electron microscopy (STEM) imaging. Because the STEM image intensity is proportional to the atomic number (I ∝ Z^1.^^7^), the FeO_x_ nanoclusters should be considerably brighter than the carbonaceous nanosheets [54]. In Fig. 1g, the bright dots represent the FeO_x_ nanoclusters, whose particle size distribution is illustrated in Fig. S10. The FeO_x_ nanoclusters were uniformly dispersed and anchored in the MIL-53(Fe) derived porous carbonaceous matrix, exhibiting narrow particle size distribution (PSD) of 9.32 ± 3.70 nm (average ± one standard deviation, S_d_) (Fig. S10) according to an evaluation of more than 300 nanoclusters from Fig. 1g and Fig. S11. The uniform distributions of C, O, and Fe in UFe-CN were also discerned in the corresponding energy-dispersive spectroscopy (EDS) mapping images (Fig. 1g), further supporting the strong interaction between the FeO_x_ nanoclusters and the graphite carbon substrate.

N_2_ adsorption-desorption isotherms (Fig. S12) were collected to assess the porous structures of UFe-CN and MIL-53(Fe). UFe-CN and MIL-53(Fe) exhibited a typical type-IV isotherm curve and a type H3 hysteresis ring, suggesting a mesoporous structure [55]. Moreover, UFe-CN exhibited a larger hysteresis ring than MIL-53(Fe), indicating that a massive mesoporous structure (Fig. S13) was formed in UFe-CN. Such porous structures benefit the overall pore diffusion for efficient PAA and other oxidant activations [56], [57], [58]. As shown in Fig. S13, the pore size distribution between 2 and 50 nm was analyzed using the Barrett-Joyner-Halenda (BJH) method, which further revealed the mesoporous structures of UFe-CN and MIL-53(Fe). Notably, the calculated average pore diameter (Table S1) of the Fe-CN catalyst (19.3 nm) was evidently larger than that of MIL-53(Fe) (6.7 nm), with the dominant size distribution at *ca.* 10 nm over that of MIL-53(Fe) at *ca.* 2 nm. Furthermore, the corresponding total pore volume of UFe-CN increased from 0.223 to 0.355 cm^3^ after carbonization, while its specific surface area decreased from 157.6 to 73.6 m ^2^ g^− 1^ (Table S1). This is due to the framework collapse of MIL-53(Fe) as well as a well-developed hierarchical porous nanoreactor that can facilitate the mass transfer. Active-site exposure can also significantly favor PAA activation for the degradation of refractory organic pollutants.

### 3.2 Promoted •OH-Dominant fenton-like process

**Fig. 2:**
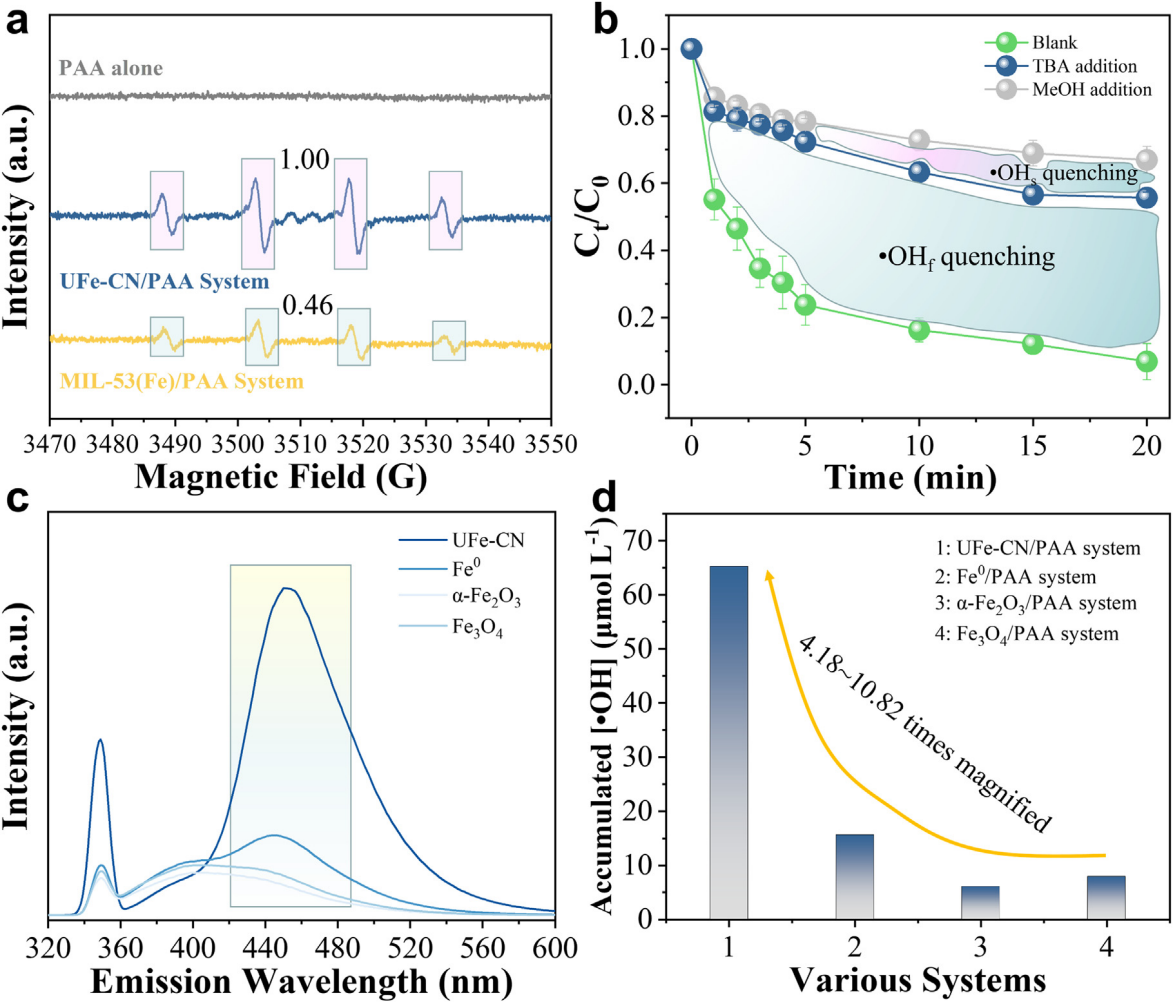
**•OH production in the PAA-based Fenton-like Processes.** (a) EPR spectra of DMPO-•OH adducts in PAA alone, UFe-CN/PAA, and MIL-53(Fe)/PAA systems. The peak intensity in the case of UFe-CN/PAA system is normalized into unit 1.00. By comparison, the peak intensity for MIL-53(Fe)/PAA system reach to 0.46. (b) 4-NP degradation efficiency in UFe-CN/PAA Fenton-like system before and after adding TBA or MeOH quenchers. (c) Fluorescence spectra of coumarin solution under different PAA activated Fenton-like systems. (d) Accumulation of •OH radicals generated by various Fenton-like systems, including UFe-CN/PAA, Fe^0^/PAA, α-Fe_2_O_3_/PAA, and Fe_3_O_4_/PAA systems. Experimental conditions: [PAA]_input_ = 0.25 mM, [DMPO] (if used) = 10 mM, [4-NP] (if used) = 20 mg *L*^−1^, [7-HC] (if used) = 10 mM, [catalyst] = 0.4 g *L*^−1^, pH_0_ = 7.0, reaction time = 20 min, *T* = 298.15 *K*.

Because the O—O peroxide bonds in PAA usually undergo various cleavages to produce ROS for organic pollutant degradation, investigating the formation of •OH and RO• (*e.g.*, CH_3_C(O)O• and CH_3_C(O)OO•), which are two typical and powerful reactive species in PAA-initiated Fenton-like processes, is important [13,59,60]. To assess the formation of •OH and RO• species via PAA decomposition, we applied the electron paramagnetic resonance (EPR) spin-trapping technique with the trapping agent DMPO, which is an effective probe for •OH and RO• species [37,59,61,62], to gain critical insights into the Fenton-like process. As illustrated in Fig. 2a, the EPR spectrum of the PAA alone system (*i.e.*, without the addition of catalysts) exhibited a flat band with no prominent peaks, suggesting the thermodynamically unavailable decomposition of PAA under the current conditions. However, the characteristic signals of the DMPO-•OH adduct (1:2:2:1, α_N_ = α_H_ = 14.9 G, *g* = 2.0055) could be easily recognized, while no indicative peaks of the DMPO-RO• adduct were detected in the EPR spectra of UFe-CN/PAA and MIL-53(Fe)/PAA systems [37,59]. This result suggests that UFe-CN can selectively decompose PAA into •OH radicals rather than R-O• radicals, as observed in many previous studies. Most importantly, the intensity of the DMPO–•OH adduct signal of UFe-CN/PAA was significantly greater than that of MIL-53(Fe)/PAA. These observations indicate that the well-designed UFe-CN heterogeneous catalyst promotes selective •OH formation rather than dividing PAA into RO• species with relatively lower oxidation potentials.

Furthermore, radical scavengers (*i.e.*, TBA and MeOH) were introduced to evaluate the contribution of •OH. TBA is effective at capturing free •OH (•OH_f_), whereas •OH_f_ and surface-bound •OH (•OH_surf_) can be easily scavenged by MeOH. Both scavengers almost completely inhibited (Fig. 2b) the degradation of 4-NP, a model refractory organic pollutant with high electron deficiency. Specifically, when TBA was used as the scavenger, the degradation rate of 4-NP decreased significantly from 93.1% to 44.3%. The residual 4-NP removal rate probably contributed to adsorption by UFe-CN as well as to degradation by other ROS species, such as superoxide radicals, whose footprint signals in the EPR spectrum were detected (Fig. S14). The addition of MeOH impeded the degradation of 4-NP by the UFe-CN/PAA system by 66.9%, which was approximately 11.2% higher than that inhibited by TBA, highlighting the marginal contribution of •OH_surf_ to 4-NP degradation. The contributions (λ) of •OH_f_, •OH_surf_, and others were calculated using Eqs. 4–6 [63], where *k_obs_* indicates the pseudo-first-order reaction rate constant of various systems. After adding TBA and MeOH, the reaction rate constants are denoted as *k*_obs_^1^ (0.061 min^−^^1^) and *k*_obs_^2^ (0.076 min^−^^1^), respectively, and the initial rate constant without scavengers is *k*_obs_^0^. •OH_f_ and •OH_surf_ radicals accounted for 76.3% and 4.7% of the contributions to the Fenton-like process, respectively, with •OH radicals accounting for a total of 81.0%. These results further support our deduction that the •OH-dominant Fenton-like process involves selective PAA decomposition facilitated by UFe-CN.

(4) $$\lambda \left(•{\text{OH}}_{\text{f}}\right)=\left[\left({k_{\mathrm{obs}}}^{0}-{k_{\mathrm{obs}}}^{1}\right)/{k_{\mathrm{obs}}}^{0}\times 100\%\right]$$

(5) $$\lambda \left(•{\text{OH}}_{\text{s}}\right)=\left[\left({k_{\mathrm{obs}}}^{0}-{k_{\mathrm{obs}}}^{2}\right)/{k_{\mathrm{obs}}}^{0}\times 100\%\right]$$

(6) $$\lambda \left(•\mathrm{OH}\right)=\lambda \left(•{\mathrm{OH}}_{f}\right)+\lambda \left(•{\mathrm{OH}}_{s}\right)$$

We further quantified the accumulated concentration of formed •OH in the proposed UFe-CN/PAA system using coumarin as a probe. Coumarin can be converted to 7‑hydroxyl coumarin (7-HC) via an oxidation reaction (Eq. 7) with 29% selectivity [64], [65], [66]. Therefore, a typical peak at an emission wavelength of *ca.* 450 nm in the fluorescence spectra (Fig. 2c) could serve as solid evidence for 7‑hydroxyl coumarin generation, implying the generation of •OH radicals. The accumulated concentration of •OH was calculated to be 65.24 µmol *L* ^−^^1^ after 15 min, which is equivalent to 26.1% utilization of PAA and considerably higher than that of other Fenton-like processes using PAA and H_2_O_2_ oxidants. Moreover, we compared the Fenton-like activity of the UFe-CN catalyst with those of other commercial benchmarks, including iron-based oxides (*i.e.*, α-Fe_2_O_3_ and Fe_3_O_4_) and zero-valent iron (Fe ^0^) (Fig. S15). The Fenton-like ability of UFe-CN to decompose PAA into •OH surpassed that of the iron-based benchmarks in terms of the accumulated •OH concentration by 4.18–10.82 times (Fig. 4d). This result suggests that the fabricated UFe-CN featuring highly conductive channels and isolated FeO_x_ nanoclusters is superior for effective PAA activation over other heterogeneous catalysts and provides encouraging prospects for treating refractory organic pollutants in various water matrices.

(7) $$\text{Coumarin}+2•\text{OH}\to 7-\text{Hydroxycoumarin}+H_{2}O$$

### 3.3 Enhanced 4-NP degradation by UFe-CN/PAA fenton-like system

**Fig. 3:**
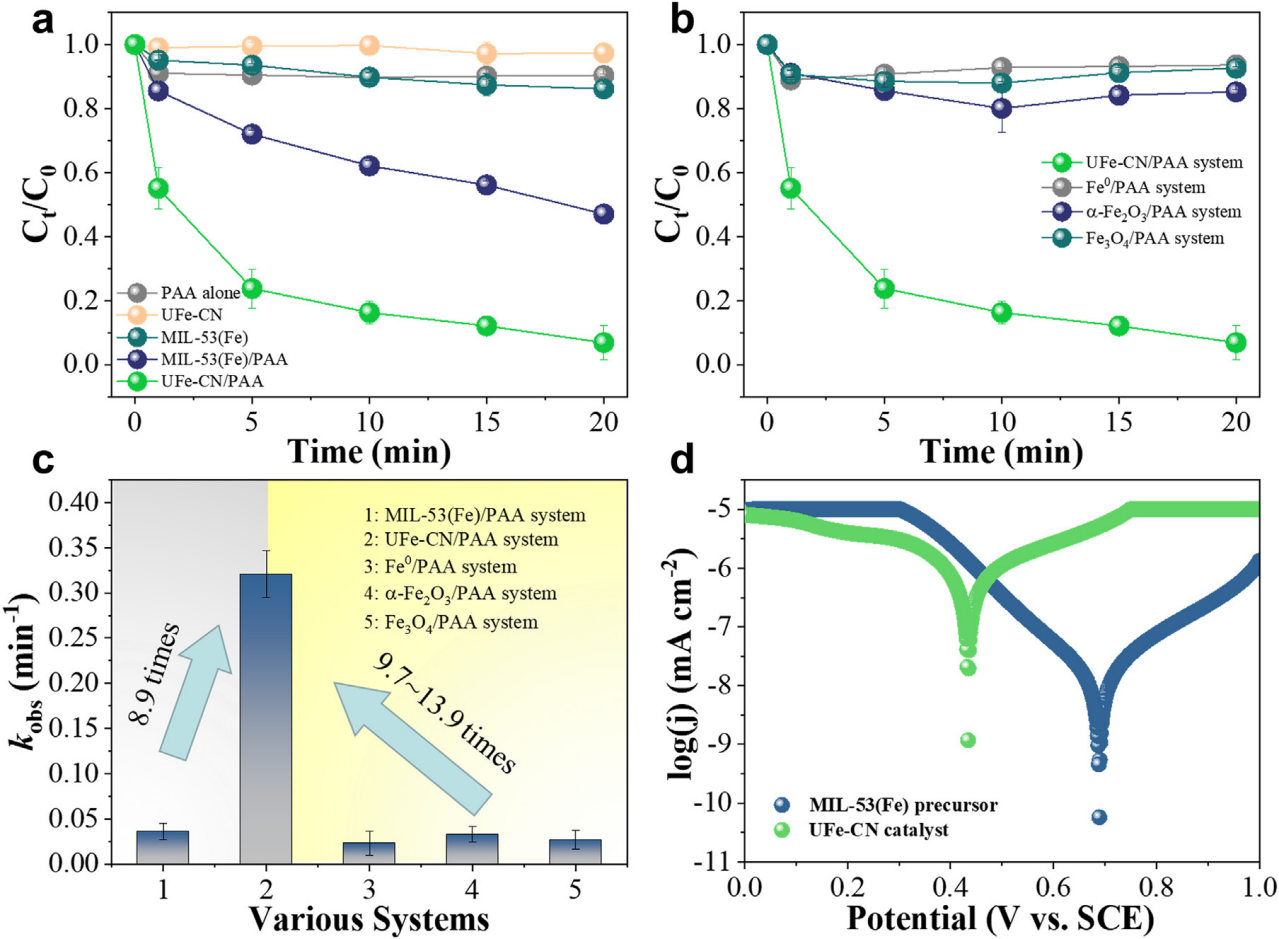
**Fenton-like reactivities of the prepared UFe-CN catalyst and other counterparts.** (a) Plots of 4-NP concentration *versus* time of UFe-CN catalyst and MIL-53(Fe) with and without PAA. (b) Plots of 4-NP concentration *versus* time in various Fenton-like systems, including UFe-CN/PAA, Fe^0^/PAA, α-Fe_2_O_3_/PAA, and Fe_3_O_4_/PAA systems. (c) *k*_obs_ values obtained from the corresponding PAA activation systems. (d) Tafel polarization curves of the prepared UFe-CN catalyst and MIL-53(Fe) precursor. Experimental conditions: [PAA]_input_ = 0.25 mM, [DMPO] (if used) = 10 mM, [4-NP] = 20 mg *L*^−1^, [catalyst] = 0.4 g *L*^−1^, pH_0_ = 7.0, reaction time = 20 min, *T* = 298.15 *K*.

The refractory organic pollutant degradation ability of the established UFe-CN/PAA Fenton-like system was systematically investigated using 4-NP as a model compound in a neutral environment owing to its high tolerance to treatments and significant toxicity to living organisms. As shown in Fig. 3a, pristine PAA contributes marginally to 4-NP degradation without adding catalysts or external energy. This means it is difficult to destroy the recalcitrant 4-NP by PAA itself, owing to the absence of highly active •OH radicals, as implied by the EPR spectrum (Fig. 2a). Moreover, UFe-CN and MIL-53(Fe) could remove 4-NP via an H-bonding-dominated adsorption process without PAA. However, the 4-NP removal efficiency of UFe-CN (2.7%) was much lower than that of MIL-53(Fe) (13.8%), owing to the significant absence of oxygen-containing groups that serve as H-bonding acceptors, as implied by the XPS analysis (Fig. 1d). However, the nanoporous UFe-CN architecture greatly favors the overall mass-transfer steps, thus improving the net degradation efficiency. In the presence of PAA, UFe-CN demonstrated a considerably high 4-NP degradation rate (93.1%) with a pseudo-first-order reaction rate constant of 0.321 min^−^^1^ (Fig. 3c), which was 8.9 times, 29.2 times, and up to 8.0 times greater than those of the MIL-53(Fe)/PAA (0.036 min^−^^1^), MIL-53(Fe)/H_2_O_2_ (0.011 min^−^^1^) and other reported Fenton-like systems (Table S2) [49]. In addition, the utilization rates of PAA for the pristine PAA, MIL-53(Fe)/PAA, and UFe-CN/PAA systems were 0.8%, 5.5%, and 29.5% (Fig. S16), respectively, using a spectrophotometric method with KI as the indicator [67].

Meanwhile, we attempted to clarify the interaction between FeO_x_ nanoclusters and the carbonaceous nanosheet matrix to improve the degradation performance by selecting Fe^0^, α-Fe_2_O_3_, and Fe_3_O_4_ as blank catalysts to trigger Fenton-like reactions. However, as shown in Fig. 3b, despite higher dosages being added to the PAA solutions than the iron compounds in UFe-CN, all the Fe^0^, α-Fe_2_O_3_, and Fe_3_O_4,_ failed to effectively degrade 4-NP, whose degradation rates only reached 6.4%, 14.8%, and 7.4% with *k*_obs_ values of 0.023 min^−^^1^, 0.033 min^−^^1^, and 0.027 min^−^^1^ (Fig. 3c), respectively. These observations (i) highlight the superior Fenton-like performance of UFe-CN for refractory organic pollutant degradation over other iron-based benchmark catalysts and (ii) emphasize the key role of the interaction between well-dispersed FeO_x_ nanoclusters and a highly conductive carbonaceous nanosheet matrix in the notably high and rapid 4-NP removal activity.

To further understand the effect of this interaction on the Fenton-like reactivity of UFe-CN, we performed electrochemical measurements to quantify electron mitigation among the prepared heterogeneous catalysts. Fig. 3d and Figs. S17-S18 disclosed the Tafel polarization curves, LSV plots, and EIS spectra of UFe-CN and MIL-53(Fe), respectively. The corrosion current value of prepared UFe-CN was calculated to be 4.56 × 10^−^^8^ A (Fig. 3d), which was 49 times higher than that of MIL-53(Fe) (9.32 × 10^−^^10^ A). The above results clarified the significantly faster electron mitigation in UFe-CN, contributing the most to the enhanced Fenton-like reactivity. In addition, a relatively stronger current density (Fig. S17) in the LSV plots and a smaller arc radius in the EIS spectrum (Fig. S18) were observed for UFe-CN compared to their counterparts. The relevant results also demonstrated rapid electron transfer between UFe-CN and PAA to improve •OH generation and 4-NP degradation [49,62,68]. These results demonstrate the significant role of such interactions in promoting electron migration for enhanced •OH generation, consequently offering promising Fenton-like reactivity for refractory organic pollutant degradation.

**Fig. 4:**
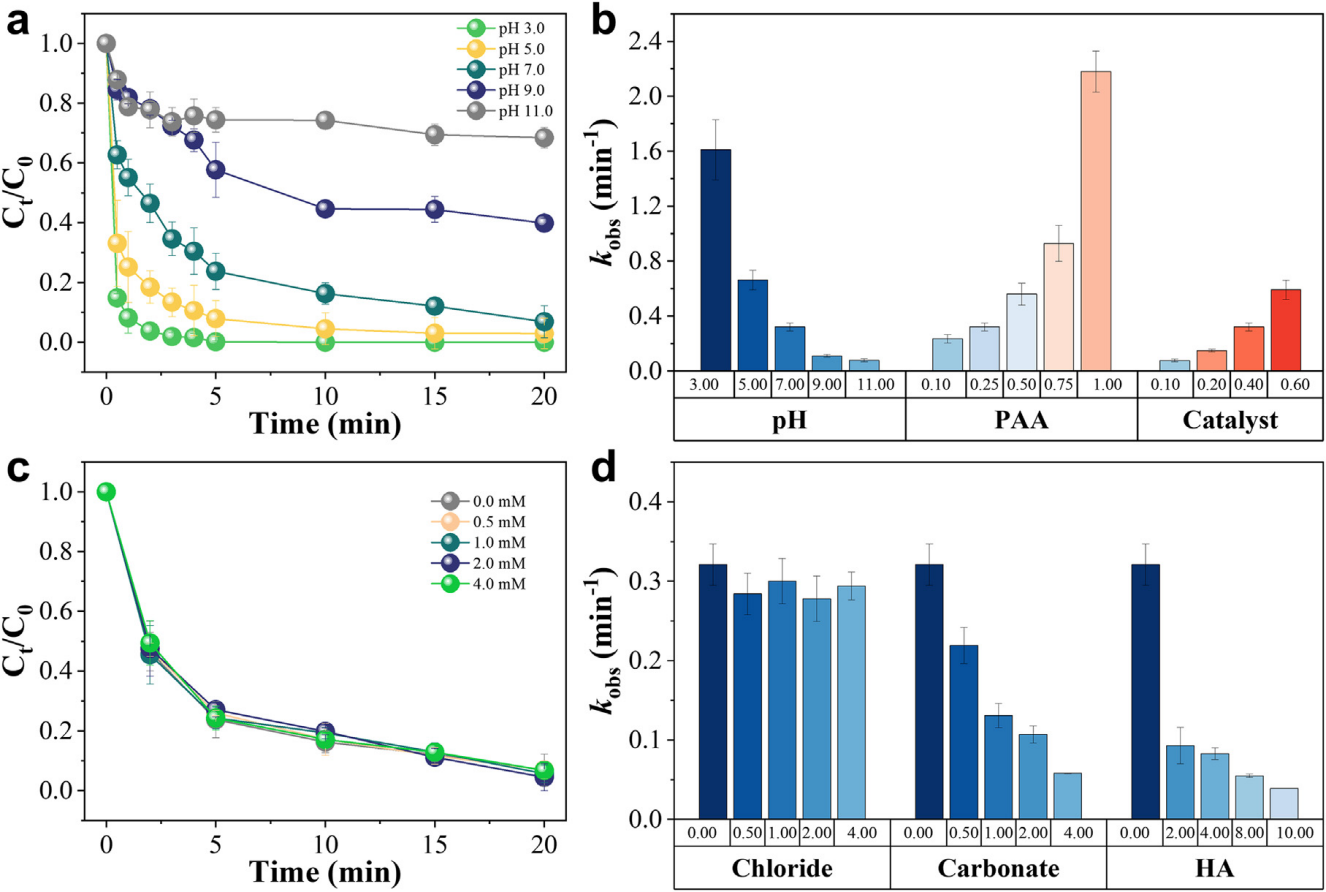
**The key reaction parameters and co-existing water matrices influencing Fenton-like reactivities of the prepared UFe-CN catalyst.** (a) Plots of 4-NP concentration *versus* time of UFe-CN catalyst at various pH_0_ ranging from 3.0 to 11.0. (b) *k*_obs_ values obtained from the UFe-CN/PAA activation systems under different reaction parameters, including pH_0_ (3.0∼11.0), PAA concentration (0.10∼1.00 mM), and catalyst dosage (0.10∼0.60 g *L*^−1^). Plots of 4-NP concentration *versus* time in various Fenton-like systems, including UFe-CN/PAA, Fe^0^/PAA, α-Fe_2_O_3_/PAA, and Fe_3_O_4_/PAA systems. (c) Plots of 4-NP concentration *versus* time of UFe-CN/PAA system with the addition of various chloride ion concentrations from 0.0 to 4.0 mM. (d) *k*_obs_ values obtained from the UFe-CN/PAA activation systems adding with different co-existing water matrices, including chloride (0.00∼4.00 mM), carbonate (0.00∼4.00 mM), and HA (0.00∼10.00 mg *L*^−1^). Experimental conditions: [PAA]_input_ = 0.25 mM, [4-NP] = 20 mg *L*^−1^, [catalyst] (unless specified) = 0.4 g *L*^−1^, pH_0_ = 7.0 (unless specified), reaction time = 20 min, *T* = 298.15 *K*.

Considering the significant importance of the Fenton-like reaction environment for adaptive engineering purposes, the tunability of UFe-CN activity under various conditions was systematically explored. The critical parameters, including the initial pH, PAA concentration, and catalyst dosage *versus* 4-NP degradation, are depicted in Fig. 4a and Figs. S19-S20. The degradation rate of 4-NP at 5 min with a pH_0_ of 3.0 decreased from 99.8% to 92.0% and 76.2% upon adjusting the pH_0_ to 5.0 and 7.0, respectively. These numbers suffered a more noticeable decline to 42.3% and 25.6% at pH_0_ of 9.0 and 11.0, respectively. The corresponding *k*_obs_ followed a similar trend, as the degradation rate decreased by nearly two orders of magnitude from 1.610 min^−^^1^ to 0.078 min^−^^1^ (Fig. 4a). The rapid decrease in the degradation rate and *k*_obs_ with pH_0_ increasing indicated that the initial pH of the UFe-CN/PAA system significantly affected the 4-NP degradation efficiency, especially under alkaline conditions (pH_0_ > 7.0). This is mainly because the hydrogen atom can easily dissociate from the PAA molecule (pKa ∼8.20) to form PAA^−^ at higher pH [44]. Dissociated hydrogen atoms are hardly adsorbed onto the surface of UFe-CN, which is driven by H-bonding interactions that potentially correlate with electrostatic repulsion [69]. Therefore, the effect of pH_0_ on 4-NP degradation followed a direction identical to that of previously reported MoS_2_/PAA [44], FeS/PAA [59], and S-ZVI/PAA [68] systems. Thus, the compelling 4-NP degradation efficiency (93.1%) in a neutral environment renders UFe-CN promising for applications.

In contrast, increasing the PAA concentration or UFe-CN dosage significantly improves the 4-NP degradation efficiency (Fig. S19). Specifically, when the concentration of PAA was increased from 0.10 mM to 1.00 mM, 4-NP could be completely removed within 5 min with a gradual increase of *k*_obs_ from 0.235 min^−^^1^ to 2.180 min^−^^1^ (Fig. 4b). Notably, when the PAA concentration declined to 0.10 mM at pH 7.0, the UFe-CN/PAA system exhibited a high 4-NP degradation rate of 73.1% (Fig. S19). The increased 4-NP degradation rate and accelerated degradation kinetics can be attributed to the production of •OH radicals at higher PAA concentrations. Furthermore, when the dosage of UFe-CN was increased to 0.6 g *L*^−^^1^, the degradation rate and *k*_obs_ reached 100% (Fig. S20) and 0.592 min^−^^1^ (Fig. 4b), respectively. Because the adsorption of 4-NP onto UFe-CN was marginal, the enhancement in 4-NP degradation was mainly attributed to the increase in the number of active sites that could accelerate the interface catalysis reaction to rapidly generate •OH radicals. We observed that the value of *k*_obs_ was linearly proportional to the input catalyst dosage (*r* > 0.99) (from 0.076 min^−^^1^ to 0.592 min^−^^1^) (Fig. S21). This observation demonstrated that the 4-NP degradation kinetics rate constant could be easily tuned, as well as validated the catalytic performance of UFe-CN for PAA activation.

### 3.4 Practical considerations of the UFe-CN/PAA Fenton-like system

To evaluate the feasibility of the UFe-CN/PAA Fenton-like system in practical applications, the tolerance of the 4-NP degradation efficiency to critical co-existing water matrices, including chloride ions, carbonate, and humic acid, was comprehensively studied. As shown in Fig. 4c, the degradation rate of 4-NP remained at a constant value of *ca.* 93% in the presence of 0–4.0 mM chloride ions, accompanied by relatively consistent *k*_obs_ values of approximately 0.3 min^−^^1^ (Fig. 4d). This insignificant impact of chloride ions suggests that the UFe-CN/PAA system exhibits high chloride resistance, proving its strong feasibility for real wastewater, especially those with high salinity [61]. However, the presence of CO_3_^2−^ (Fig. S22) and HA (Fig. S23) largely inhibited the 4-NP degradation even at a low concentration of 0.5 mM and 4 mg L^−^^1^, respectively. In particular, increasing the concentration of CO_3_^2−^ (from 0 mM to 4.0 mM) or HA (from 0 mg *L*^−^^1^ to 40 mg *L*^−^^1^) can further reduce the 4-NP degradation kinetic rate constant by one order of magnitude (Fig. 4d). The negative effect of carbonate may have been caused by the consumption of •OH by carbonate, as shown in Eq. 8 [70]. However, the phenolic hydroxyl and carboxyl groups of HA are adsorbed onto the catalyst and block the active sites on its surface, inhibiting PAA activation [37]. Moreover, HA can capture •OH radicals on the UFe-CN surface and/or in aqueous solutions.

(8) $$\mathrm{OH}+{{\mathrm{CO}}_{3}}^{2-}\to •C{O_{3}}^{-}+{\mathrm{OH}}^{-}k=3.2\times 108\;M^{-1}\;s^{-1}$$

**Fig. 5:**
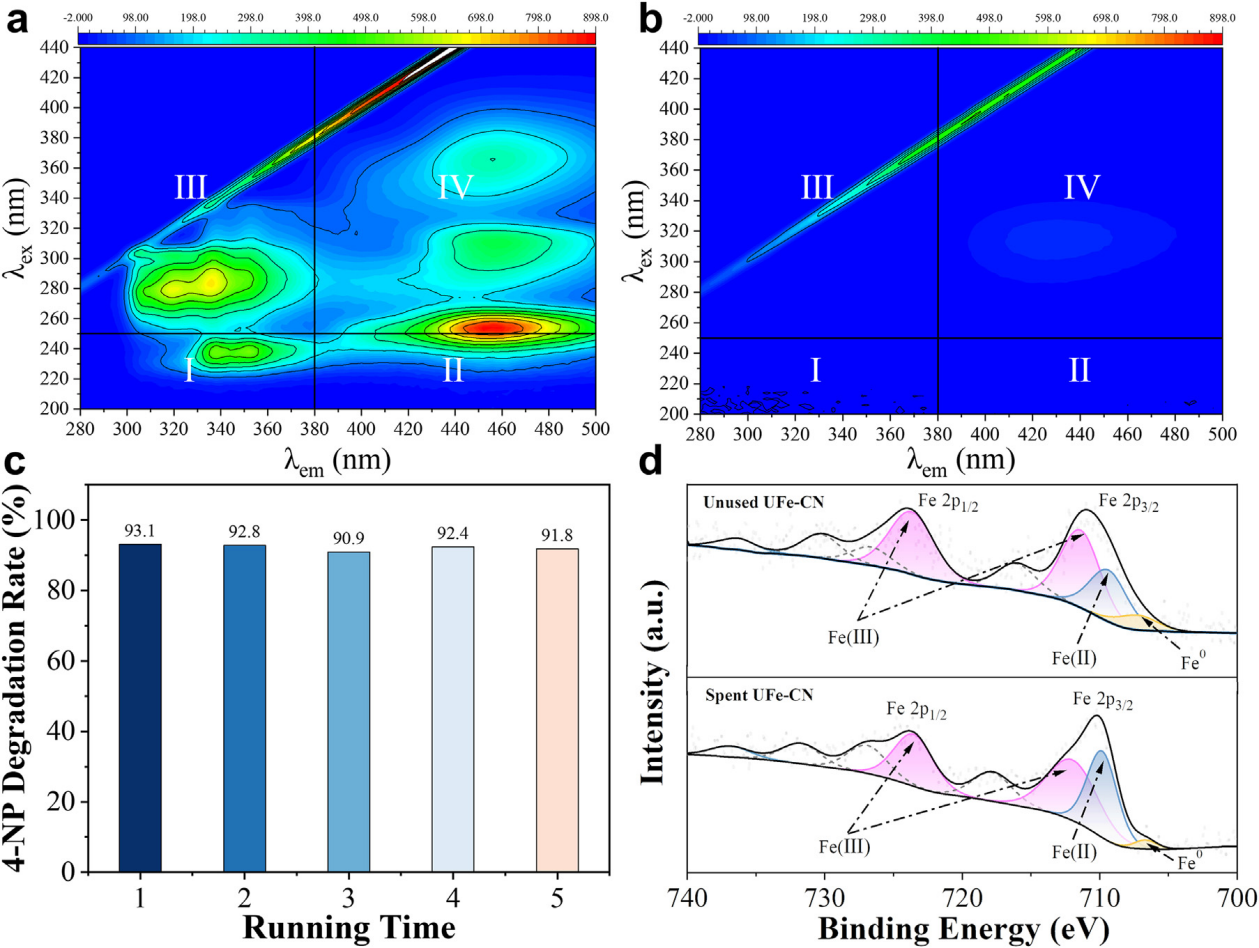
**Practicability considerations of the UFe-CN/PAA Fenton-like system.** (a) 3D EEM spectra of the collected TD&F wastewater before (a) and after (b) treated by the UFe-CN/PAA Fenton-like system. (c) 4-NP degradation rate *versus* successive running times of 5. (d) High-solution Fe 2p XPS spectra of the unused and spent UFe-CN catalysts. Experimental conditions: [PAA]_input_ = 0.25 mM, [4-NP] (if used) = 20 mg L^−1^, [catalyst] = 0.4 g L^−1^, V for TD&F wastewater = 50 mL, pH_0_ = 7.0, reaction time = 20 min, *T* = 298.15 K.

To further examine the practicality of the UFe-CN/PAA Fenton-like system, we collected textile dyeing and finishing (TD&F) wastewater with abundant phenolic compounds for treatment. Owing to the complex composition of TD&F wastewater, we recorded its 3D EEM spectra before and after treatment using the UFe-CN/PAA system. As shown in Fig. 5a, the possible fluorescent substances in the collected sample were classified as simple aromatic proteins, fulvic acid-like compounds, soluble microbial byproduct analogs, and humic acid-like organics, which could be easily identified by the specific regions of I (λ_ex_/λ_em_ = (200–250 nm)/(280–380 nm)), II (λ_ex_/λ_em_ = (200–250 nm)/(380–550 nm)), III (λ_ex_/λ_em_ = (> 250 nm)/(< 380 nm)), and Ⅳ (λ_ex_/λ_em_ = (> 280 nm)/(> 380 nm)), respectively [46]. According to the above classification, Sample A contained all four types of organic matter. The relatively stronger intensities of regions I, II, and III suggest that this sample contained more aromatic compounds than humic acid-like organics. After treatment with UFe-CN/PAA for 20 min, the organic pollutants in the wastewater were entirely removed, as indicated by the disappearance of the footprint peaks (Fig. 5b). Owing to the divergent components of the TD&F wastewater at various periods, we also treated another TD&F wastewater (*i.e.*, sample B). As expected, the organic pollutants in sample B were also eliminated by the UFe-CN/PAA system after 60 min (Fig. S24), indicating that UFe-CN could effectively activate PAA for wastewater decontamination with considerable potential.

In addition, the reusability and stability of UFe-CN in PAA activation processes were investigated to evaluate its potential in engineering operations. As illustrated in Fig. 5c, the catalytic performance of UFe-CN was maintained at a high level, with a 4-NP degradation rate greater than 90.0% after five consecutive cycling runs. Typically, the corresponding *k*_obs_ in the 5th run reached 0.299 min^−^^1^ (Fig. S25), which was nearly consistent with the performance of the 1st run. Meanwhile, the leaching amount of iron was less than 5 mg L^−1^ (Fig. S26), equal to no more than 1.25 wt% loss of the catalysts in each cycle. Moreover, the leaching amount after the 3rd cycle conformed to the discharge standard proposed by the European Union and the textile wastewater reuse standard (NO. FZ/T 01,107–2011) issued by the Ministry of Industry and Information Technology (China) [46]. More importantly, the spent UFe-CN showed no apparent changes in morphology, crystal configuration, or chemical structure compared to its pristine counterpart according to the FESEM (Fig. S27), PXRD (Fig. S28), and FT-IR (Fig. S29) measurements. However, the chemical composition of the spent UFe-CN showed a slight increase in Fe(II) along with the reduction of Fe(III) and almost vanishing Fe^0^ (Fig. 5d). Because the excellent cycling of the Fe(II)/Fe(III) redox couples favors •OH generation and improves the Fenton-like reactivity, the ratio of Fe(II)/Fe(III) in UFe-CN before and after use were calculated (Figs. 1d and 5d). Particularly, as shown in Table S3, the Fe(II)/Fe(III) ratio on the surface of UFe-CN reduced from 0.52 to 0.51 after usage, suggesting that the UFe-CN has an excellent Fe(II)/Fe(III) redox cycle property for efficient PAA activation. According to previous studies [8,13], Fe(II) easily changes to Fe(III) after the activation of PAA. Subsequently, Fe(II) is likely to be reduced to Fe(II) species through one-electron transfer from the organic pollutant and/or carbonaceous matrix, which is believed to serve as an electron donor for the heterogeneous Fenton-like process [71], [72], [73]. Motivated by these results, the prepared UFe-CN could be an alternative to the highly effective PAA-based Fenton-like processes.

## 4 Conclusion

We demonstrated a novel and efficient heterogeneous PAA activation system for refractory phenolic compound degradation by incorporating a rationally designed UFe-CN. The ultrafine FeO_x_ nanocluster component was spatially separated to expose more active iron sites, whereas the carbonaceous nanosheets served as a highly conductive electron-transfer network to facilitate interfacial Fenton-like catalysis reactions. The UFe-CN can alter the PAA activation pathway from R-O• to •OH dominant process, enhancing refractory and toxic 4-NP degradation under a wide pH range (3.0–11.0). The Fenton-like reactivity of UFe-CN outperformed its benchmark iron-based counterparts by 4.2–10.8 times, which was mainly driven by the significantly enhanced electron mitigation. Furthermore, the performance can be easily tuned by varying the key engineering parameters such as the initial pH, PAA concentration, and catalyst dosage to fulfill adaptive engineering aims. This study provides ideas for the construction of highly efficient PAA activation systems and significant insights into the promotion of •OH-dominant PAA-based Fenton-like processes.

## Declaration of competing interest

The authors declare that they have no conflicts of interest in this work.

## Appendix B Supplementary materials

Image, application 1
