# Supplementary material for: Promoting •OH-dominant Fenton-like process over peracetic acid activation by ultrafine FeOx nanoclusters anchored carbonaceous nanosheets
Source: Fundam Res. 2023 May 30;5(6):2580–90. doi: 10.1016/j.fmre.2023.05.007 (PMC12744666; doi:10.1016/j.fmre.2023.05.007)
Supplement: Supplementary file 1 [file mmc1.docx]

**Supporting Information**

**Promoting** •**OH-Dominant Fenton-like Process over Peracetic Acid Activation by Ultrafine FeO_x_ Nanoclusters Anchored Carbonaceous Nanosheets**

Qian Hu^a, b^, Taoyu Yang^a^, Shanli Wang^a^, Licong Xu^a, c^, Minghua Wu^a^, Deyou Yu^a, c^*, Kaixing Fu^b^, Jinming Luo^b^*

^a^ Engineering Research Center for Eco-Dyeing and Finishing of Textiles (Ministry of Education), College of Textile Science and Engineering, Zhejiang Sci-Tech University, Hangzhou 310018, PR China

^b^ School of Environmental Science and Engineering, Shanghai Jiao Tong University, Shanghai 200240, PR China

^c^ Zheijiang Sci-Tech University Tongxiang Research Institute, Tongxiang, 345000, PR China

* Corresponding authors:

Deyou Yu, Email: [yudeyou92@zstu.edu.cn](mailto:yudeyou92@zstu.edu.cn)

Jinming Luo, Email: [jinming.luo@sjtu.edu.cn](mailto:jinming.luo@sjtu.edu.cn)

2 texts

27 figures

3 tables

11 references

**Text S1. Quantification of the accumulated concentration of •OH.**

We first plotted the standard curve of 7-hydroxyl coumarin concentration versus fluorescence intensity. The resultant equation is determined to be “Intensity = 37.7*[7-HOC] (R^2^ ˃ 0.999)”. Then, we measured the intensity of withdrawn samples, whose 7-hydroxyl coumarin (7-HOC) concentration could be easily determined by the obtained equation. At last, we calculated the accumulated concentration of •OH by following equation.

[•OH] = 2*[7-HOC]/Se

where [7-HOC] and Se indicate the concentration of 7-HOC and the selectivity, respectively.

**Text S2. Determination of the corrosion current.**

Corrosion current values referring to Tafel polarization curves were calculated by an extrapolation method. The main process is to do the tangent linear fitting of anode polarization and cathode polarization, and the x-axis corresponding to the intersection of the two tangent lines denote as log(i/A), where i is the corrosion current value while A is the electrode area. Therefore, the calculation equation of corrosion current is i = πr2 ×10^log(i/A), where r is 0.15 cm in our measurement.


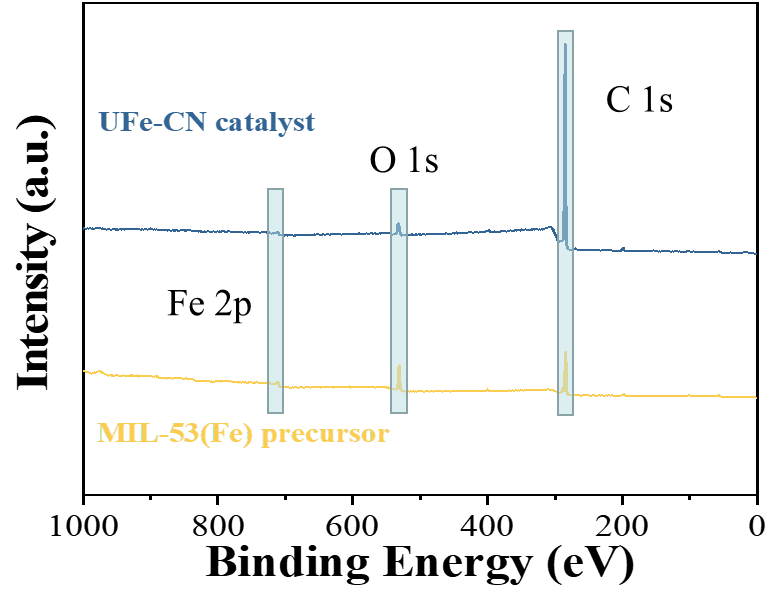


Figure S1. XPS elemental survey of UFe-CN catalyst and MIL-53(Fe) precursor.


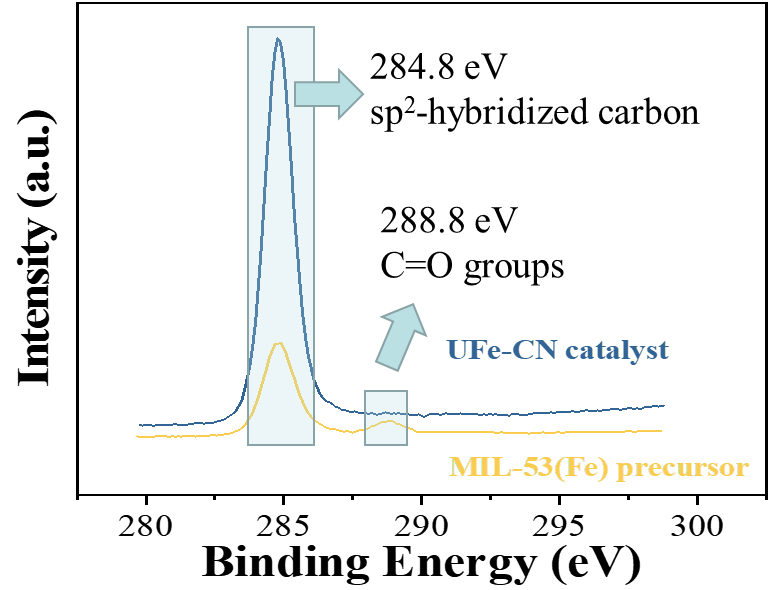


Figure S2. High-resolution C 1s XPS spectra of UFe-CN catalyst and MIL-53(Fe) precursor. Two peaks locating at 284.8 and 288.8 eV refer to the sp^2^-hybridized carbon and C=O groups, respectively.


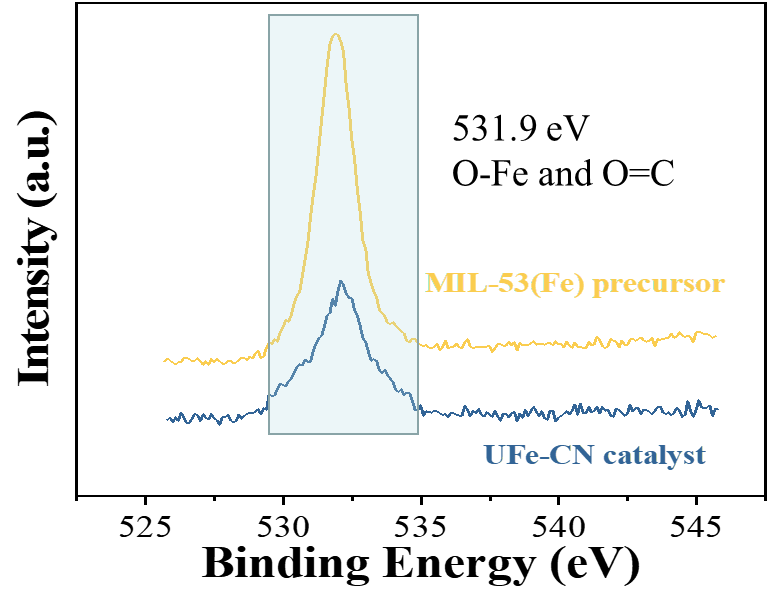


Figure S3. High-resolution O 1s XPS spectra of UFe-CN catalyst and MIL-53(Fe) precursor. The main peak locating at 531.9 eV indicates the overlapping of the O-Fe and O=C groups.





Figure S4. Surface morphology revealed by FESEM image of the prepared UFe-CN catalyst, where FeO_x_ nanoclusters could be easily found on the rough surface of graphite matrix.


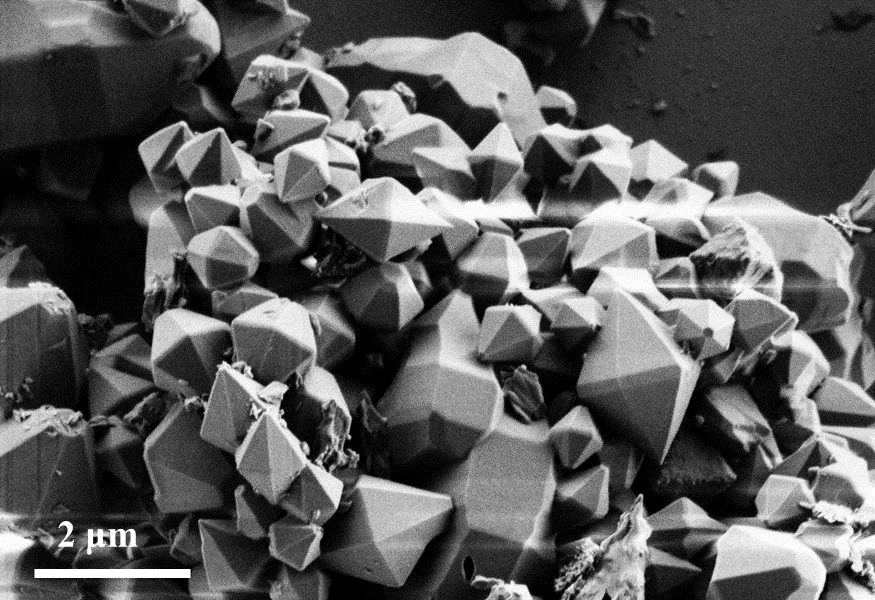


Figure S5. Morphology revealed by FESEM image of the MIL-53(Fe) precursor, where a smooth regular dodecahedron crystalline shape could by easily found.


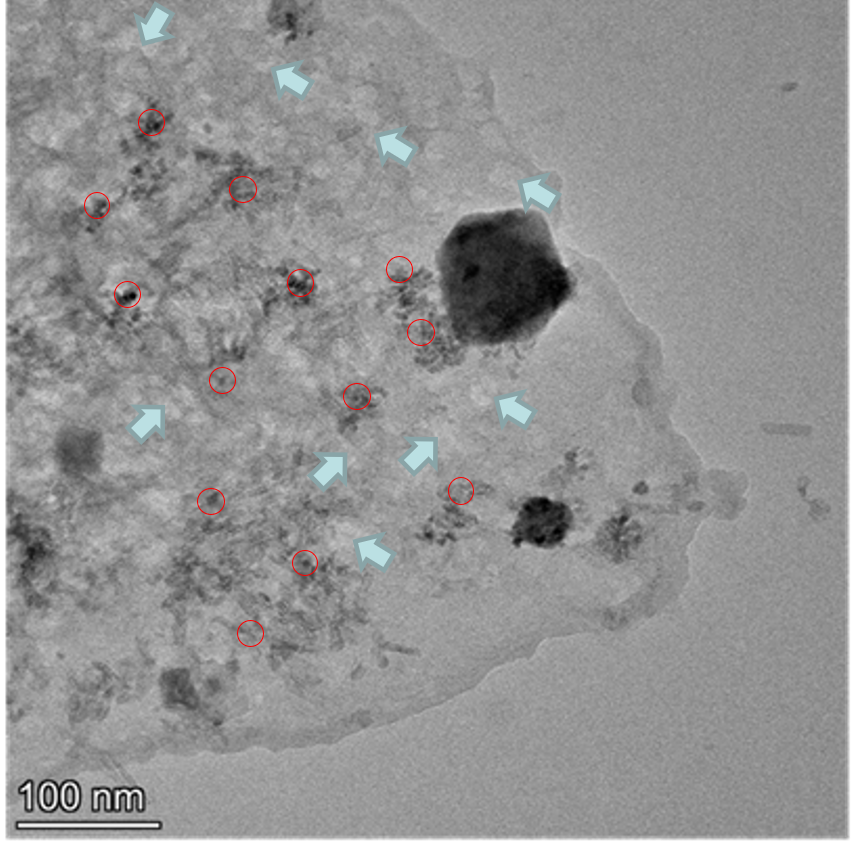


Figure S6. Further confirmation of UFe-CN catalyst’s nanosheet construction by TEM image with larger magnification than Figure 1f. Red circles and blue arrows indicate the FeOx nanoclusters and nanoporous channels.


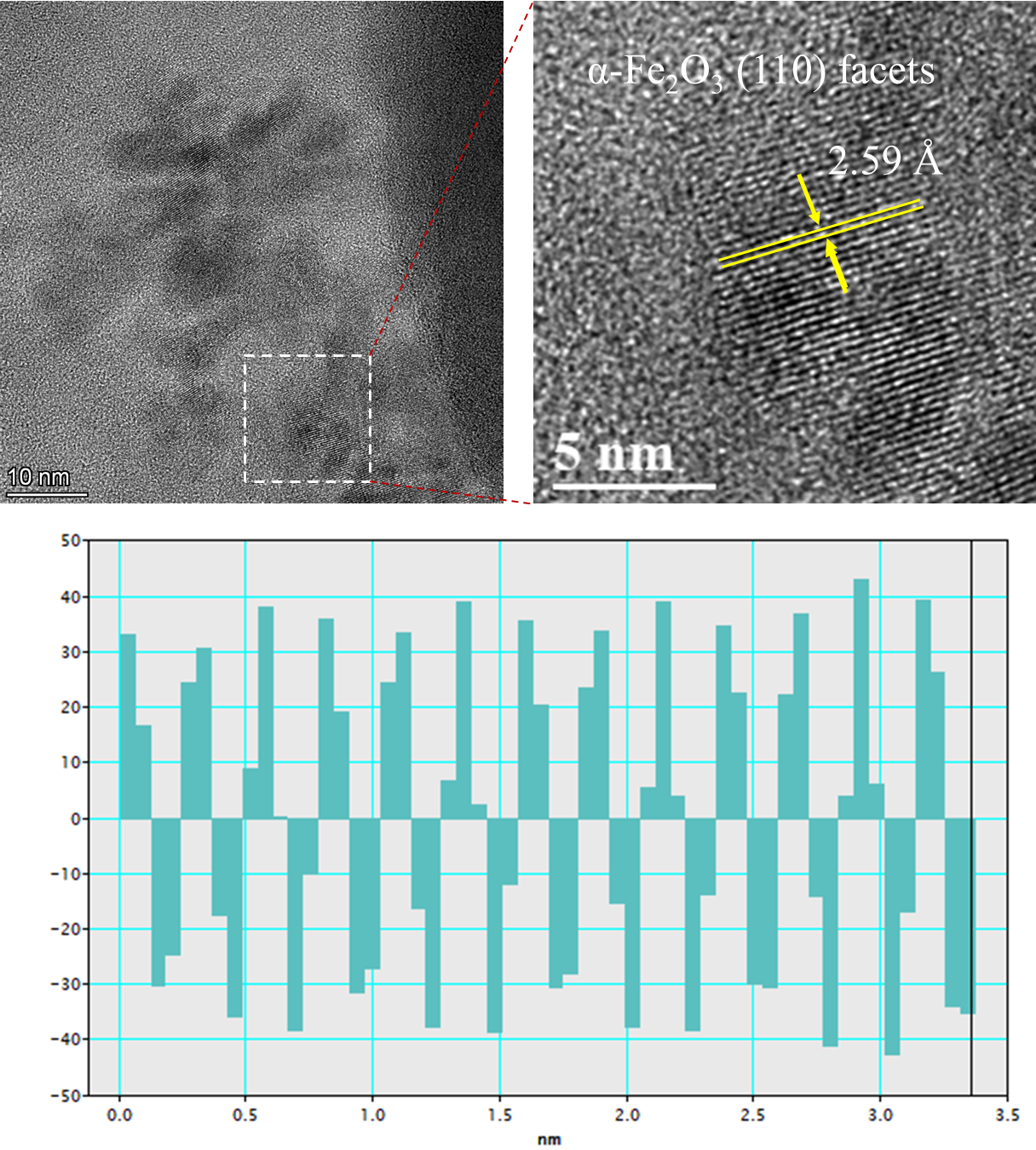


Figure S7. High resolution transmission electron microscopy images and grayscale profile of the prepared UFe-CN catalyst, in which a typical d-spacing value of 2.59 Å representing α-Fe_2_O_3_ (110) facets could be observed.


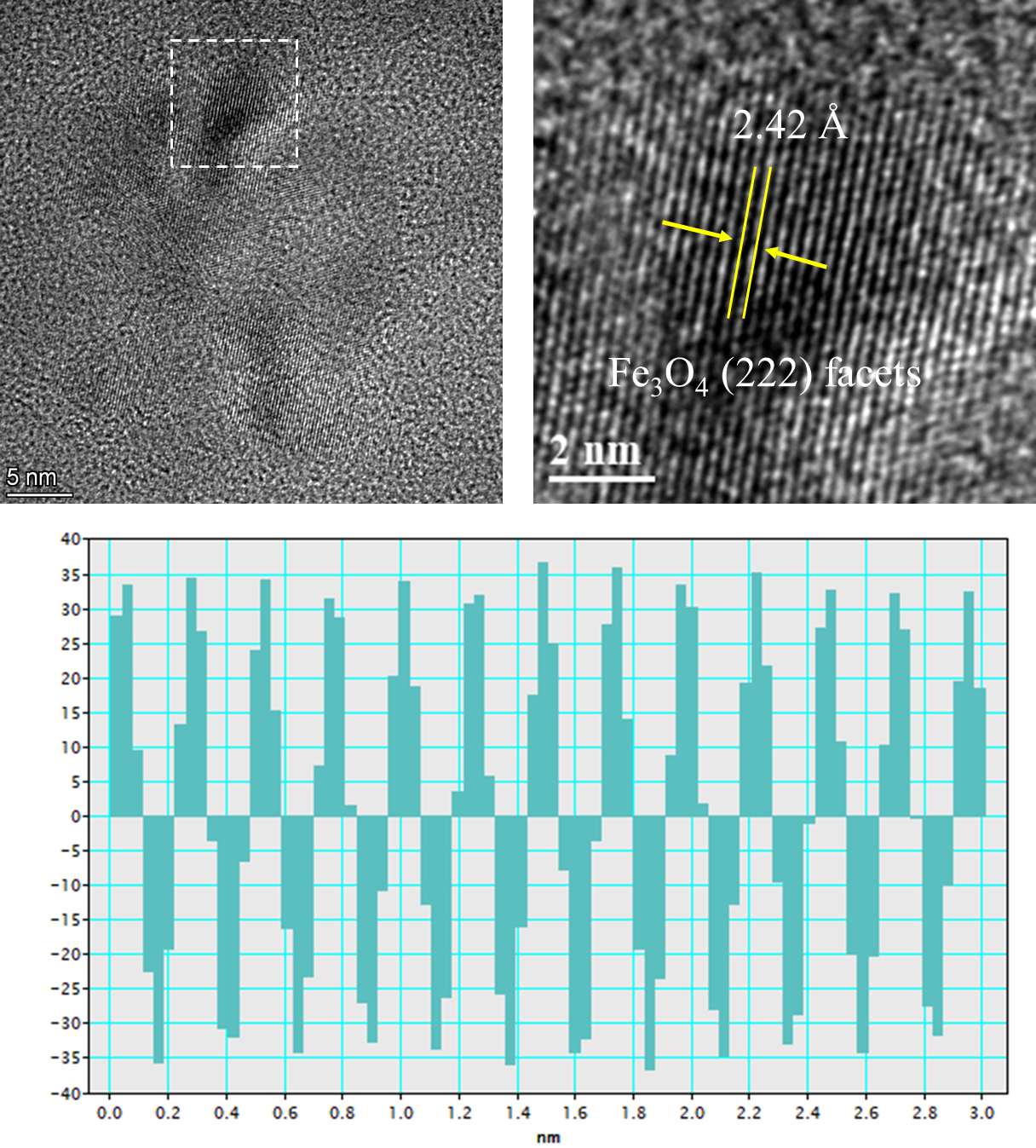


Figure S8. High resolution transmission electron microscopy images and grayscale profile of the prepared UFe-CN catalyst, in which a typical d-spacing value of 2.42 Å representing Fe_3_O_4_ (222) facets could be observed.


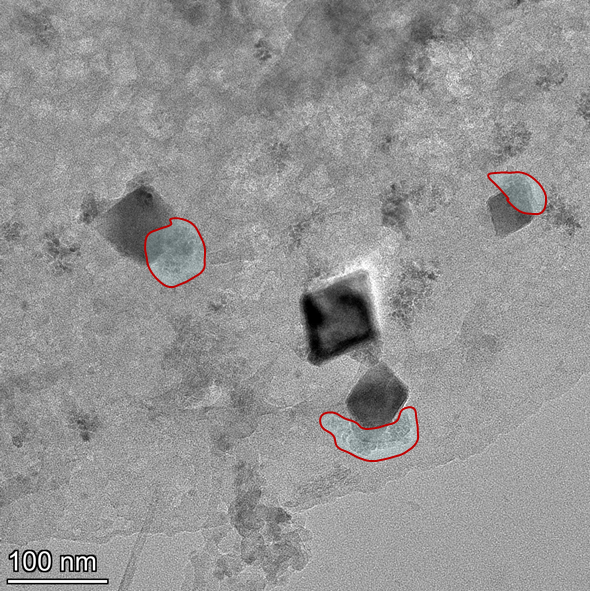


Figure S9. TEM image of the prepared UFe-CN catalyst with local magnifications. Red circle demonstrates the possibility that the FeO_x_ nanoclusters are derived from the collapse of the MIL-53(Fe) precursor.


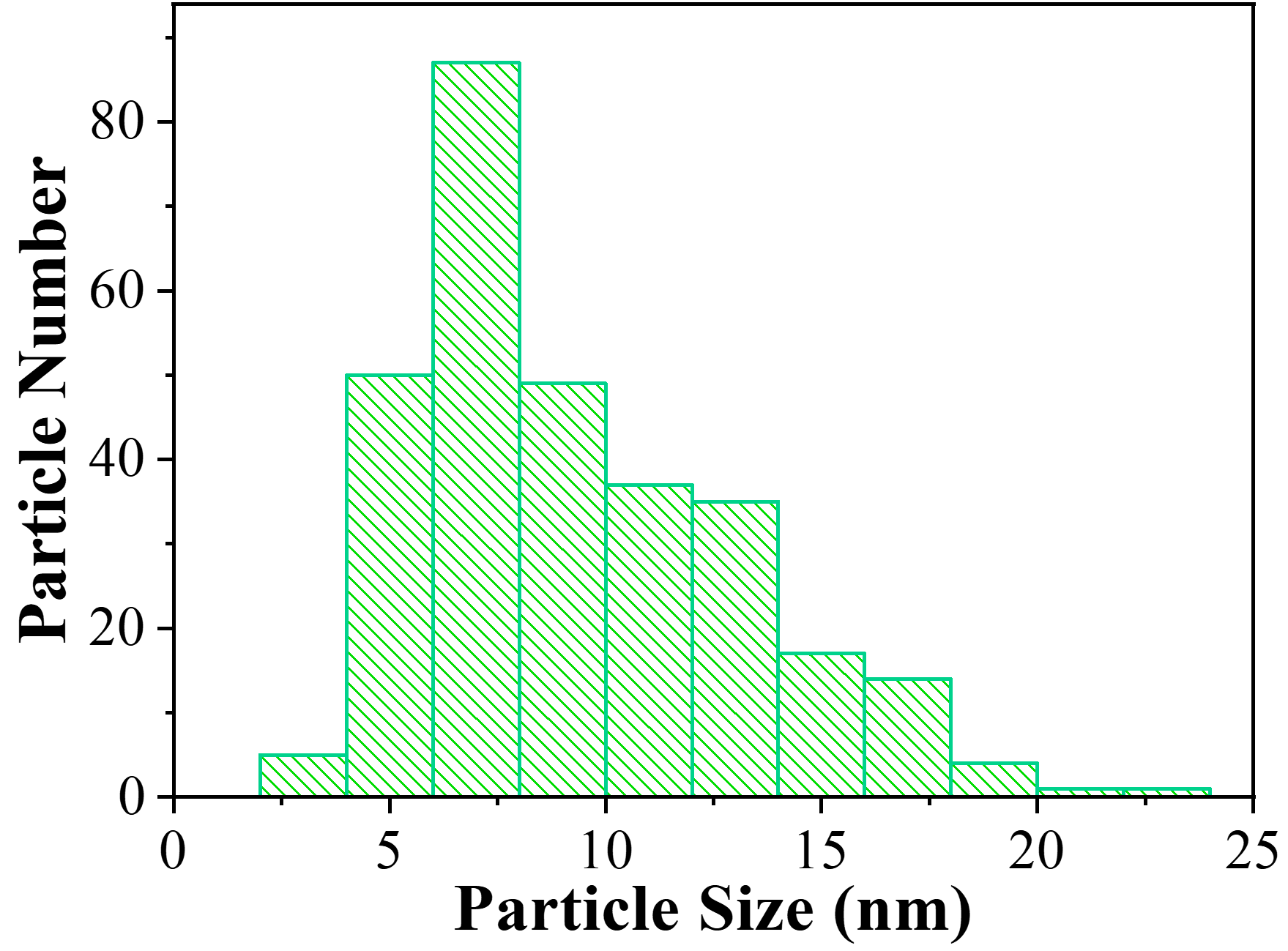


Figure S10. Particle size distribution (PSD) histogram of about 300 nanoclusters analyzed from Figure 2e and Figure S10.


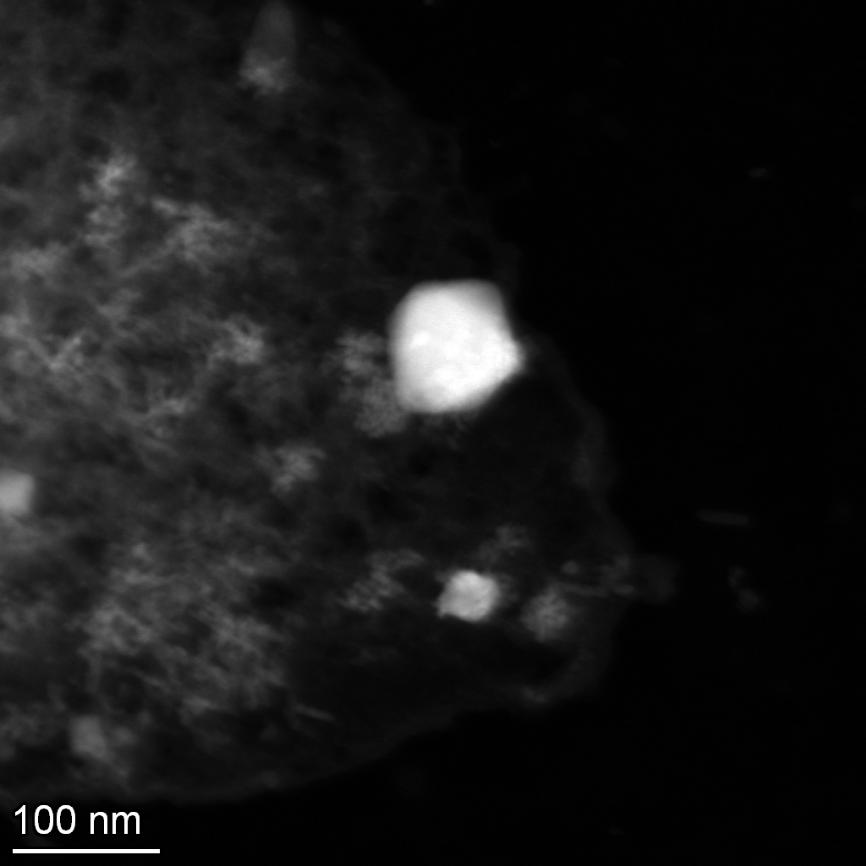


Figure S11. Collection of low-magnification STEM image of FeO_x_ nanoclusters anchored in a MOF-derived porous carbon matrix (scale bar, 100 nm). Around 300 nanoclusters were counted and analyzed for the PSD histogram in Figure. S9. Very few particles larger than 25 nm were not included in the PSD histogram.


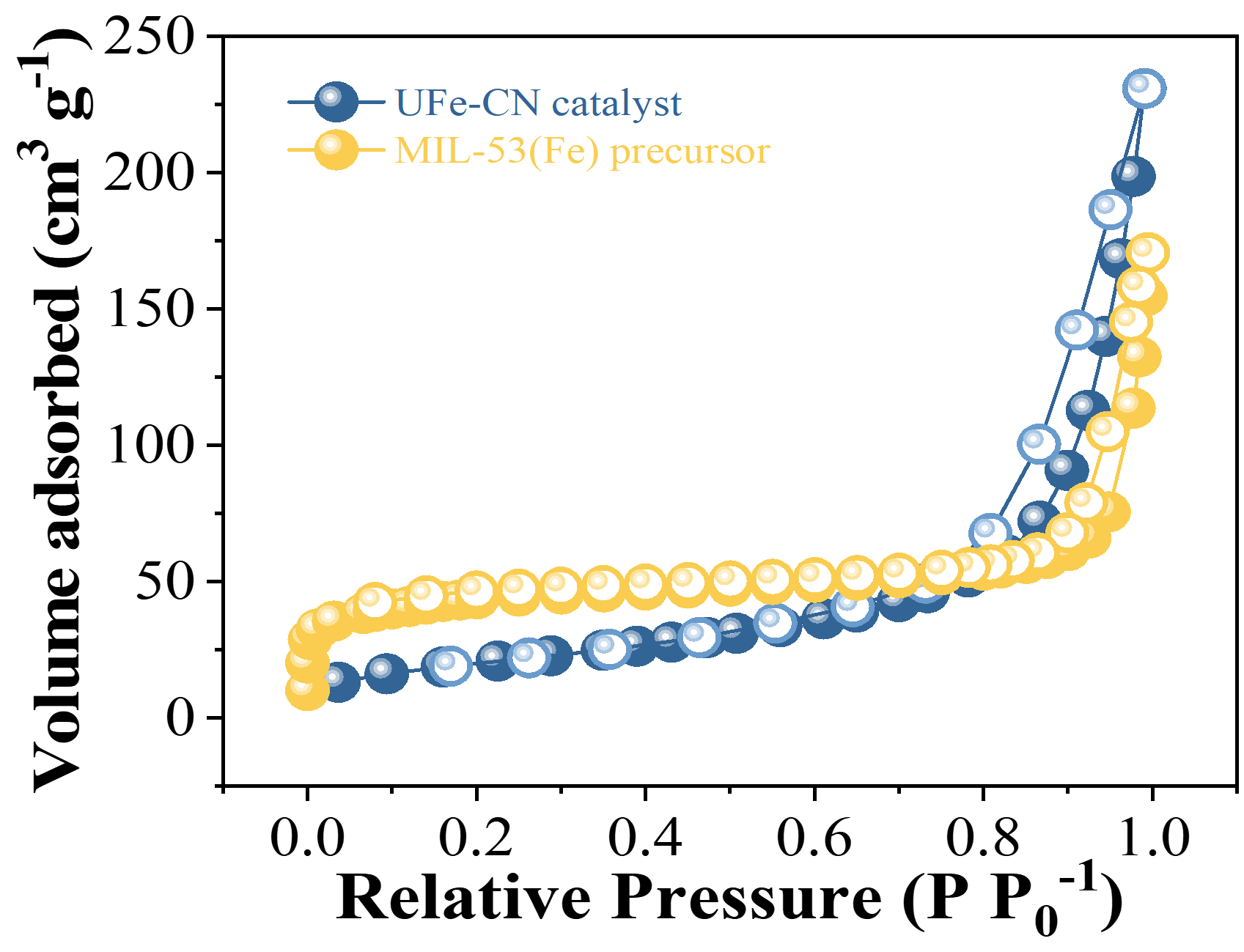


Figure S12. N_2_ adsorption-desorption of UFe-CN catalyst and MIL-53(Fe) precursor.


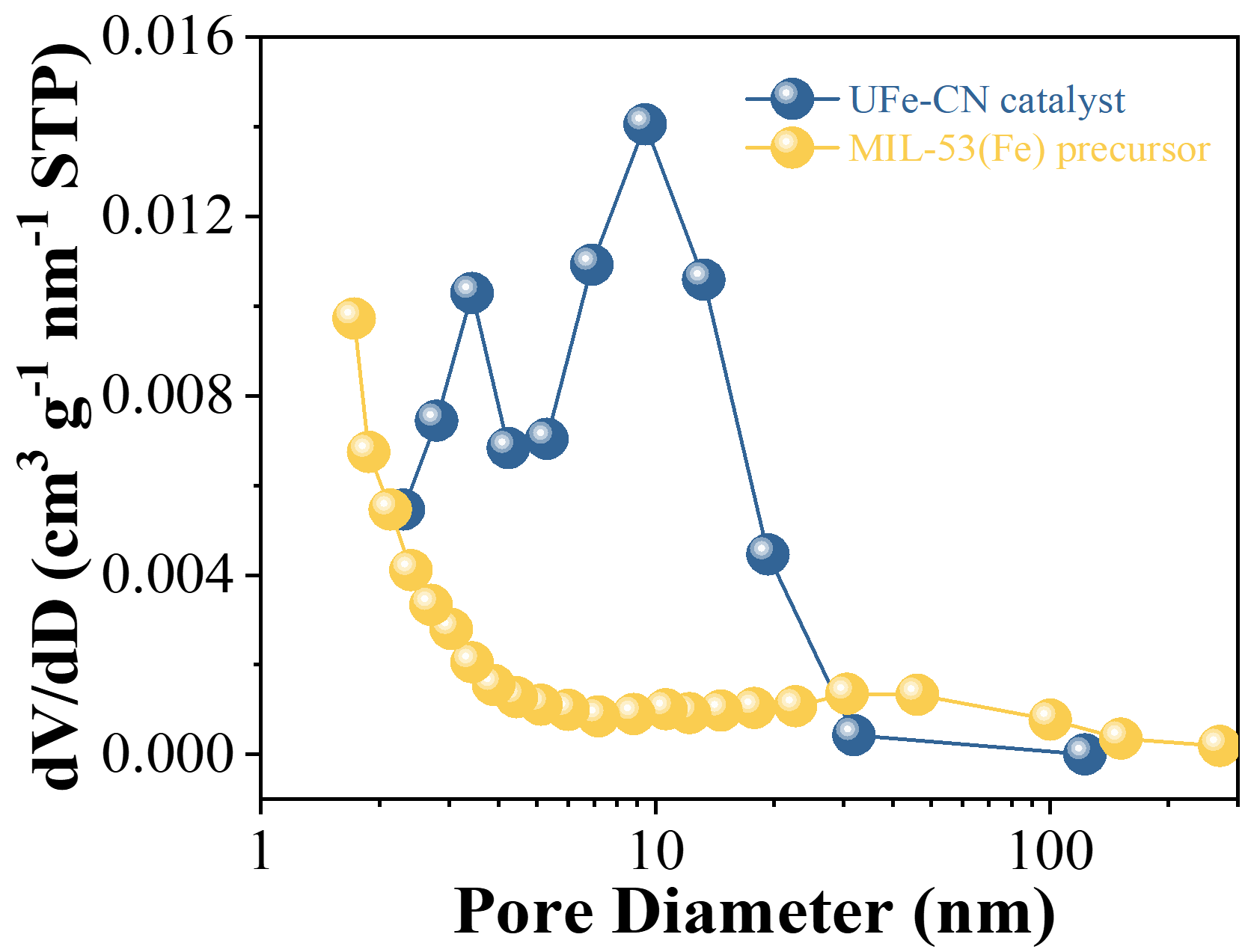


Figure S13. Pore diameter distribution plots of UFe-CN catalyst and MIL-53(Fe) precursor.


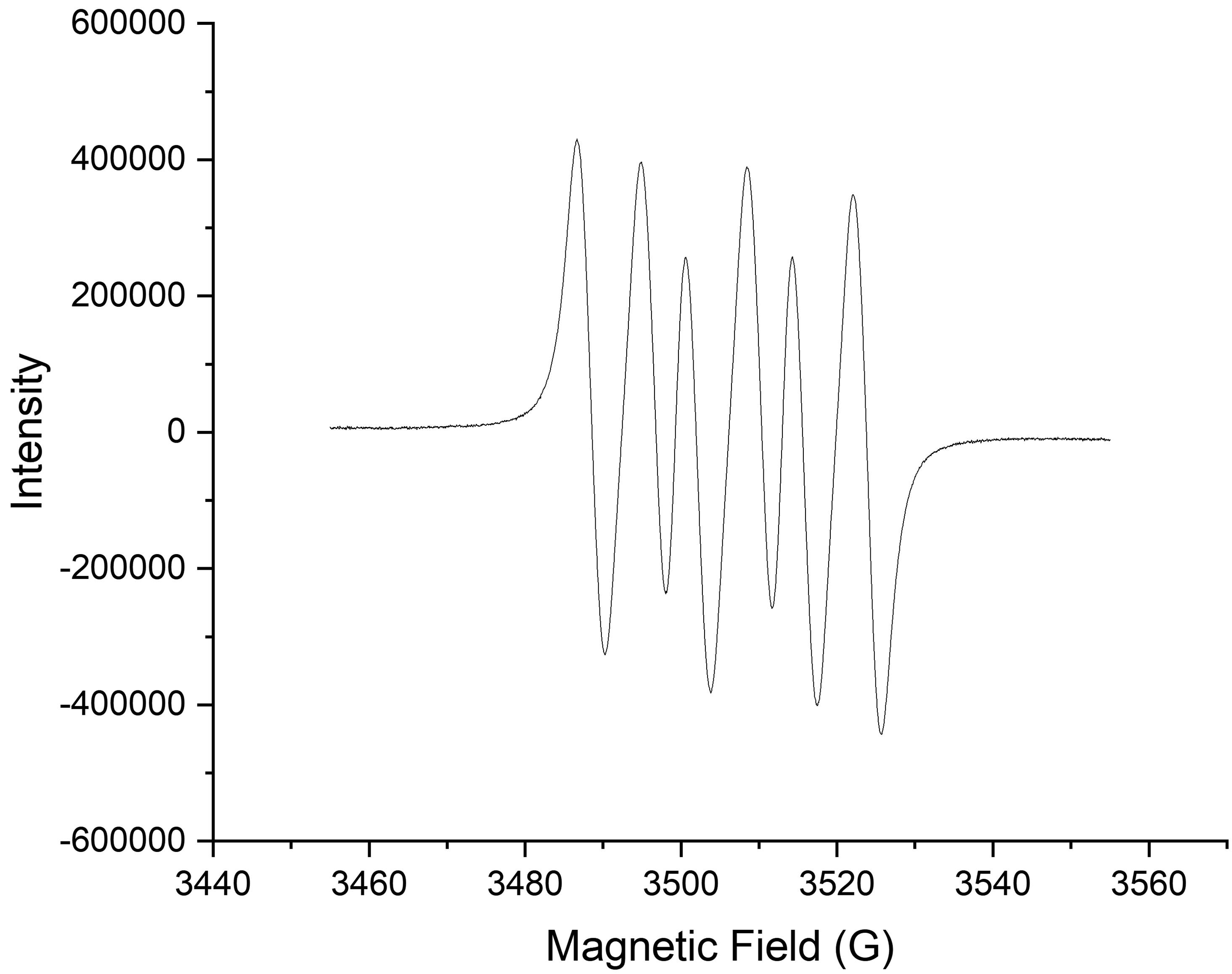


Figure S14. EPR spectrum of DMPO-•O_2_^-^ adducts in UFe-CN/PAA activation system.


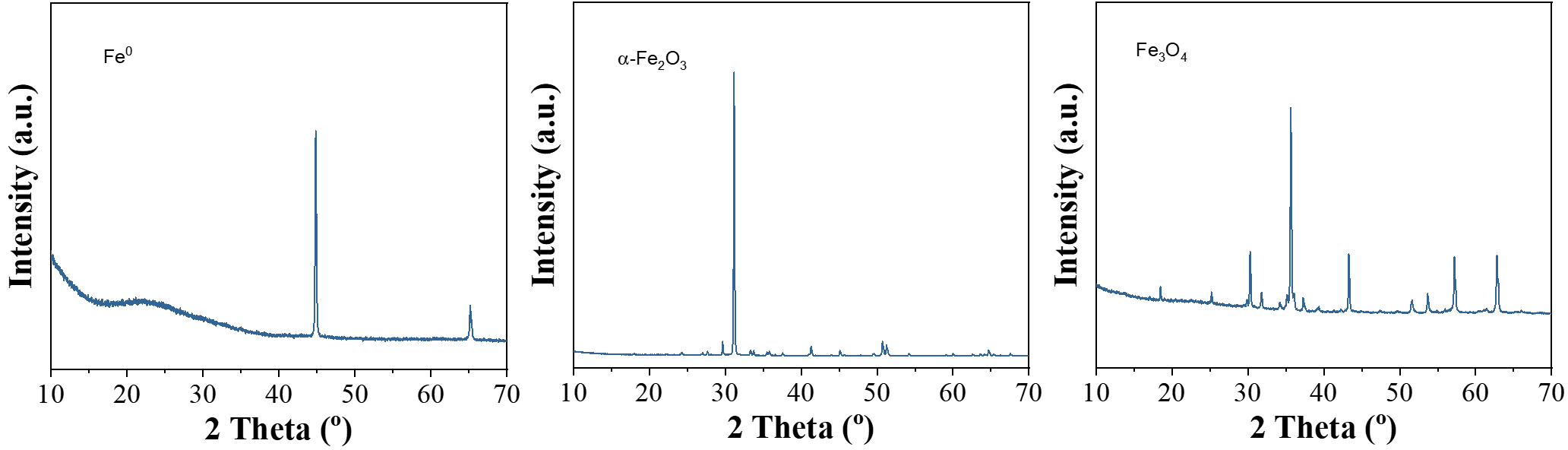


Figure S15. PXRD patterns of the received commercial Fe^0^, α-Fe_2_O_3_, and Fe_3_O_4_ benchmarks.


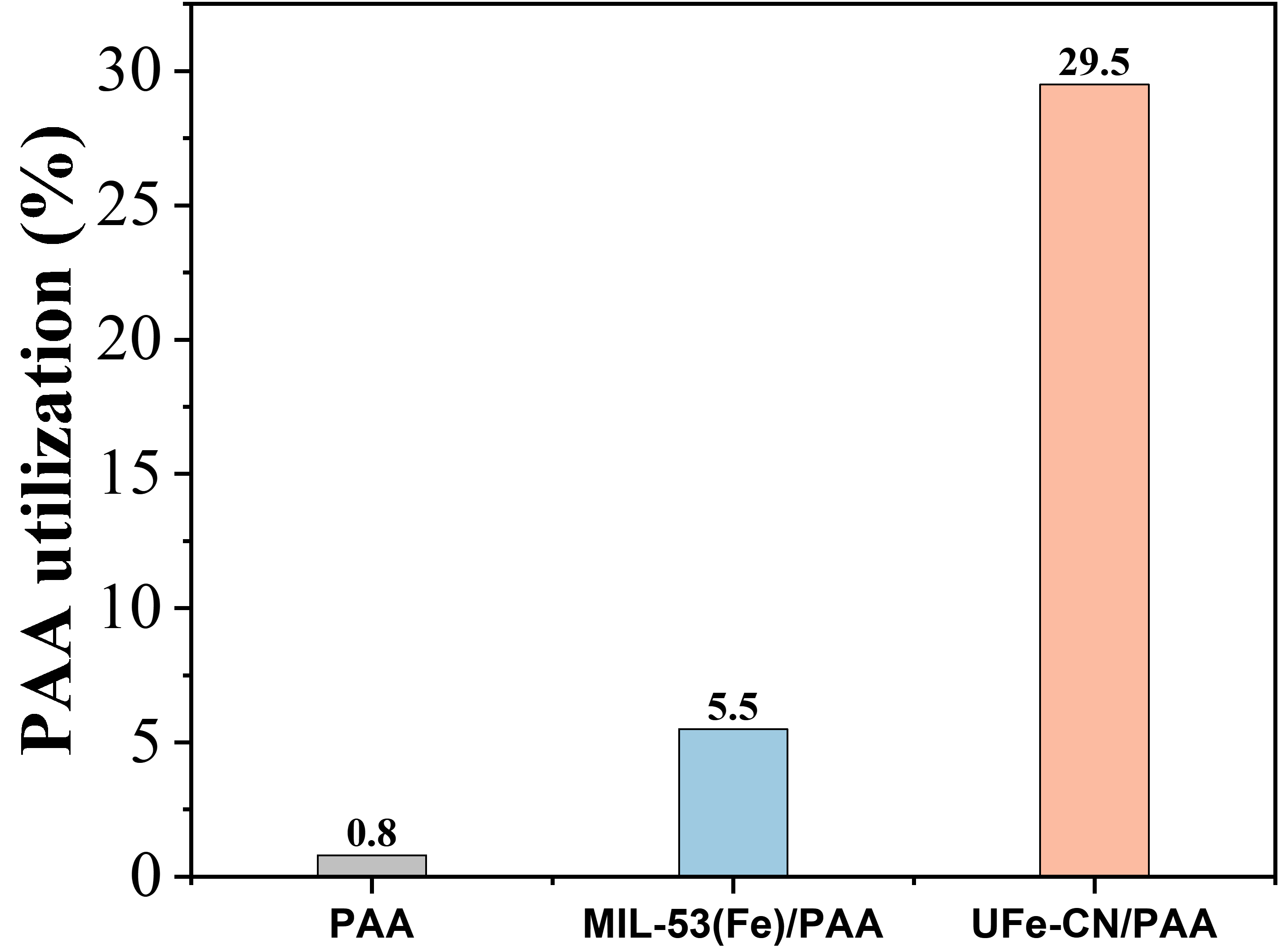


Figure S16. PAA utilization for PAA alone, MIL-53(Fe)/PAA, and UFe-CN/PAA systems.


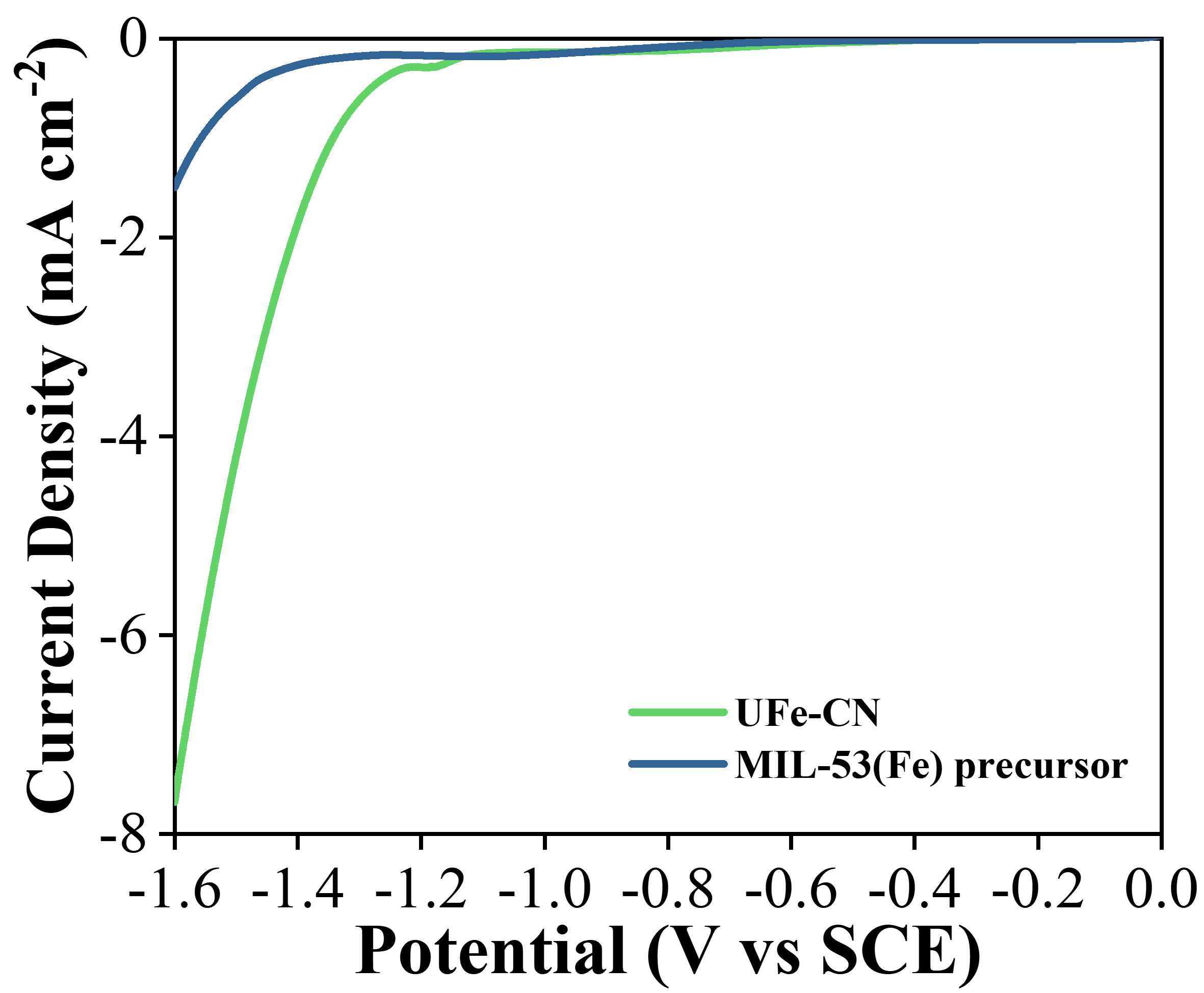


Figure S17. LSV plots of the prepared UFe-CN catalyst and MIL-53(Fe) precursor. The UFe-CN catalyst gets a current density of about -1.53 mA cm^-2^ while it is -7.73 mA cm^-2^ for MIL-53(Fe) precursor.


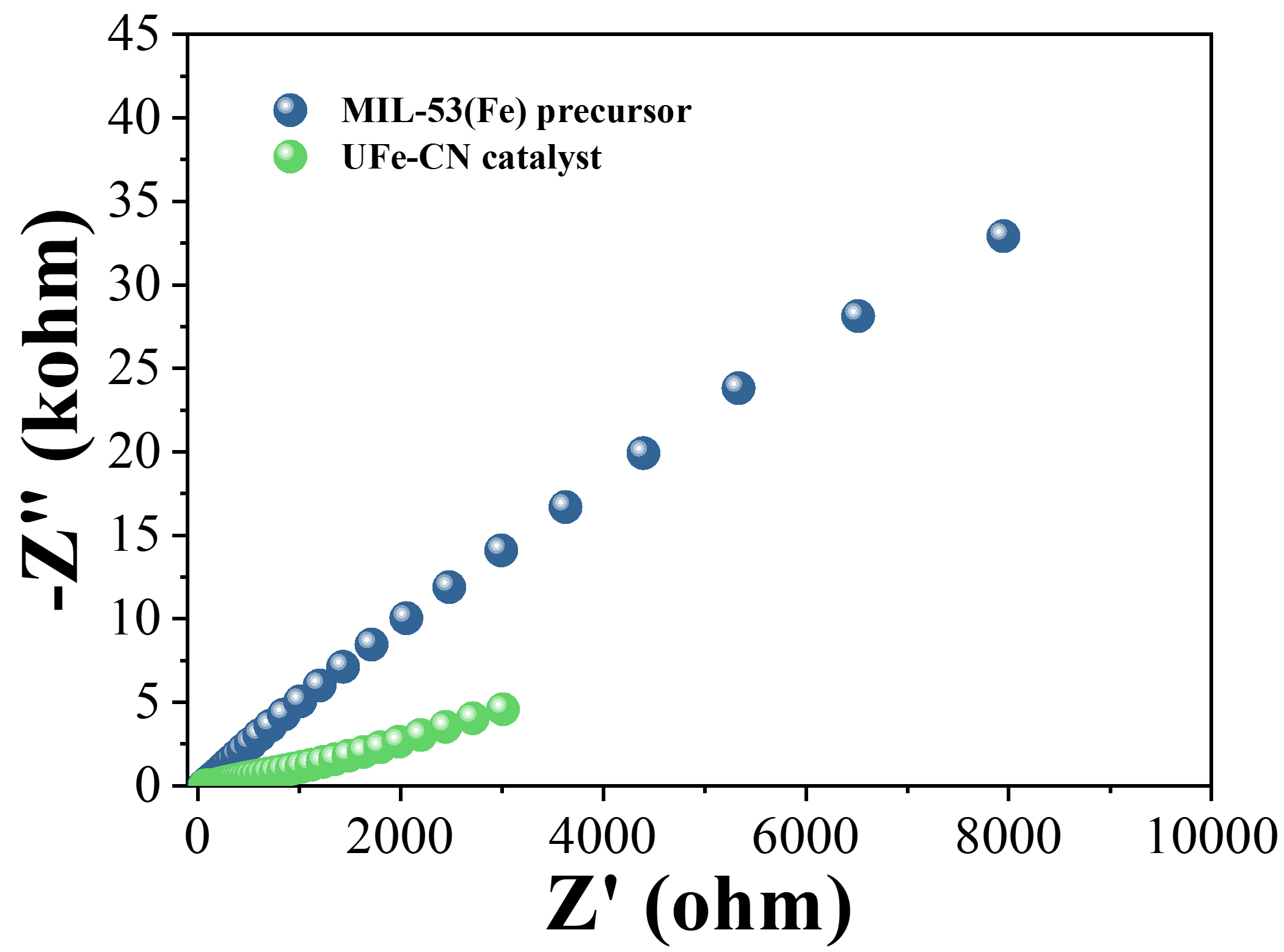


Figure S18. EIS spectra of the prepared UFe-CN catalyst and MIL-53(Fe) precursor.


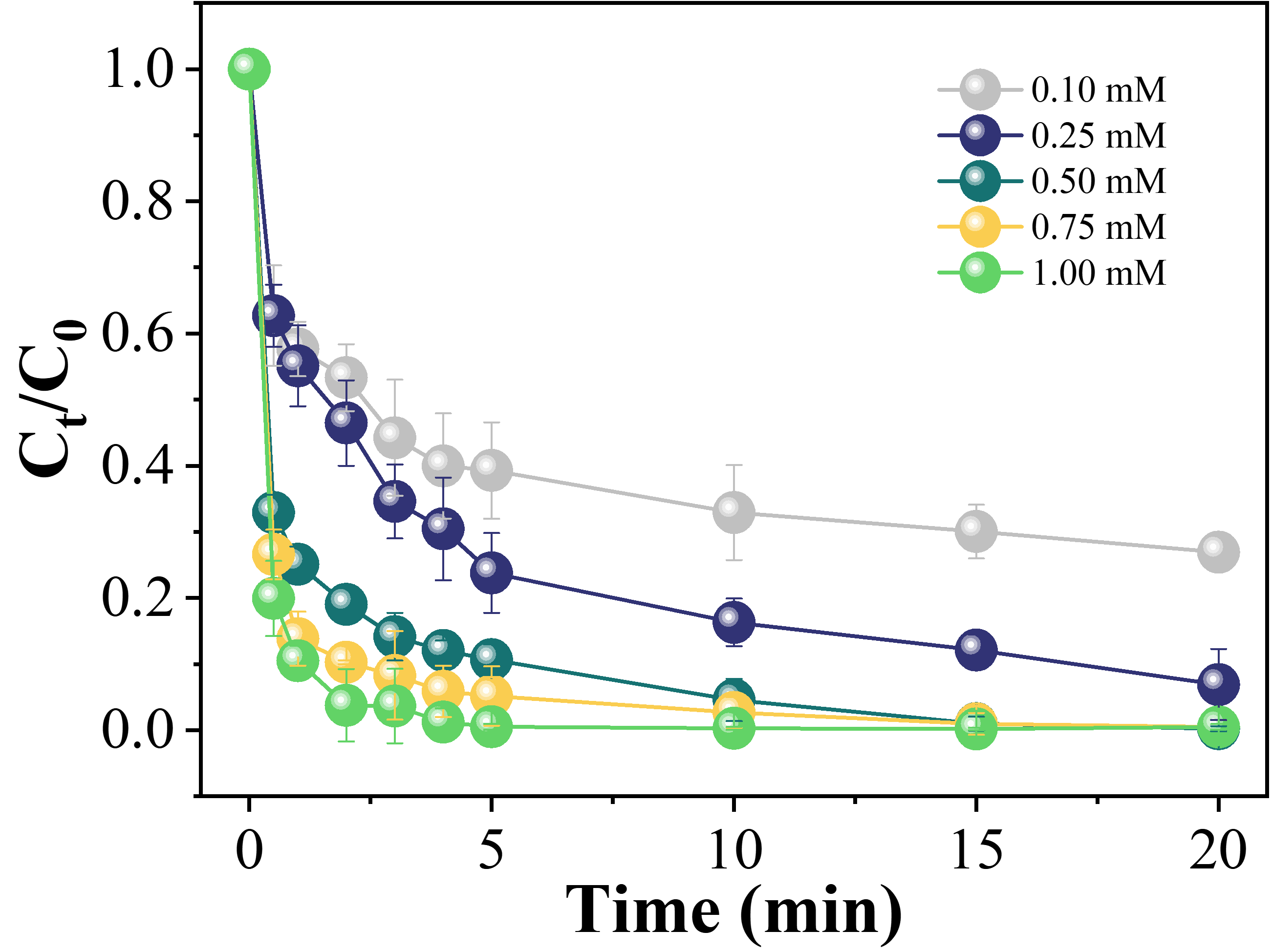


Figure S19. 4-NP degradation in the UFe-CN/PAA Fenton-like system at different PAA concentrations. Experimental conditions: [4-NP] = 20 mg L^-1^, [catalyst] = 0.4 g L^-1^, pH_0_ = 7.0, reaction time = 20 min, T = 298.15 K.


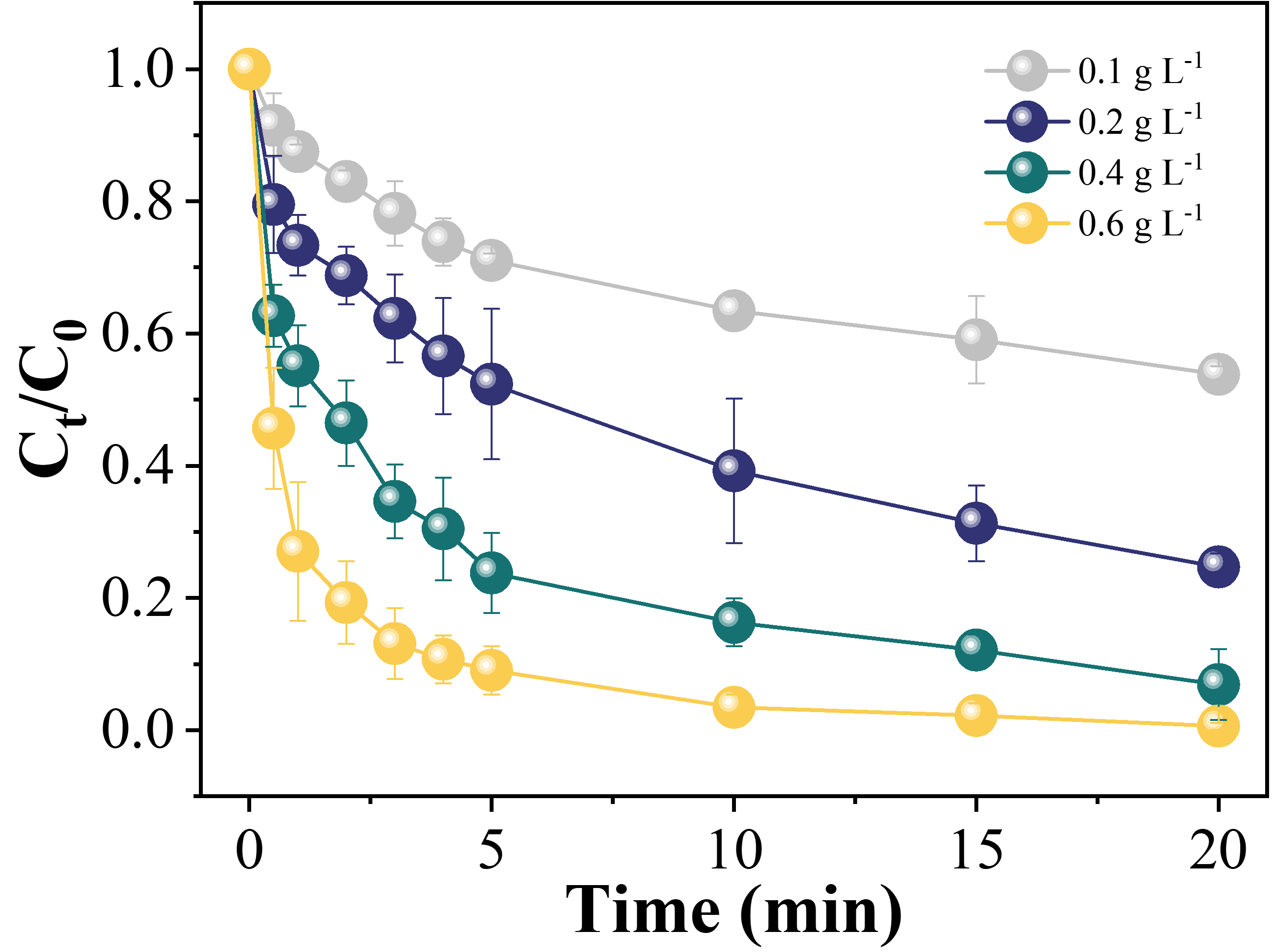


Figure S20. 4-NP degradation in the UFe-CN/PAA Fenton-like system at different catalyst dosages. Experimental conditions: [4-NP] = 20 mg L^-1^, [PAA] = 0.25 mM, pH_0_ = 7.0, reaction time = 20 min, T = 298.15 K.


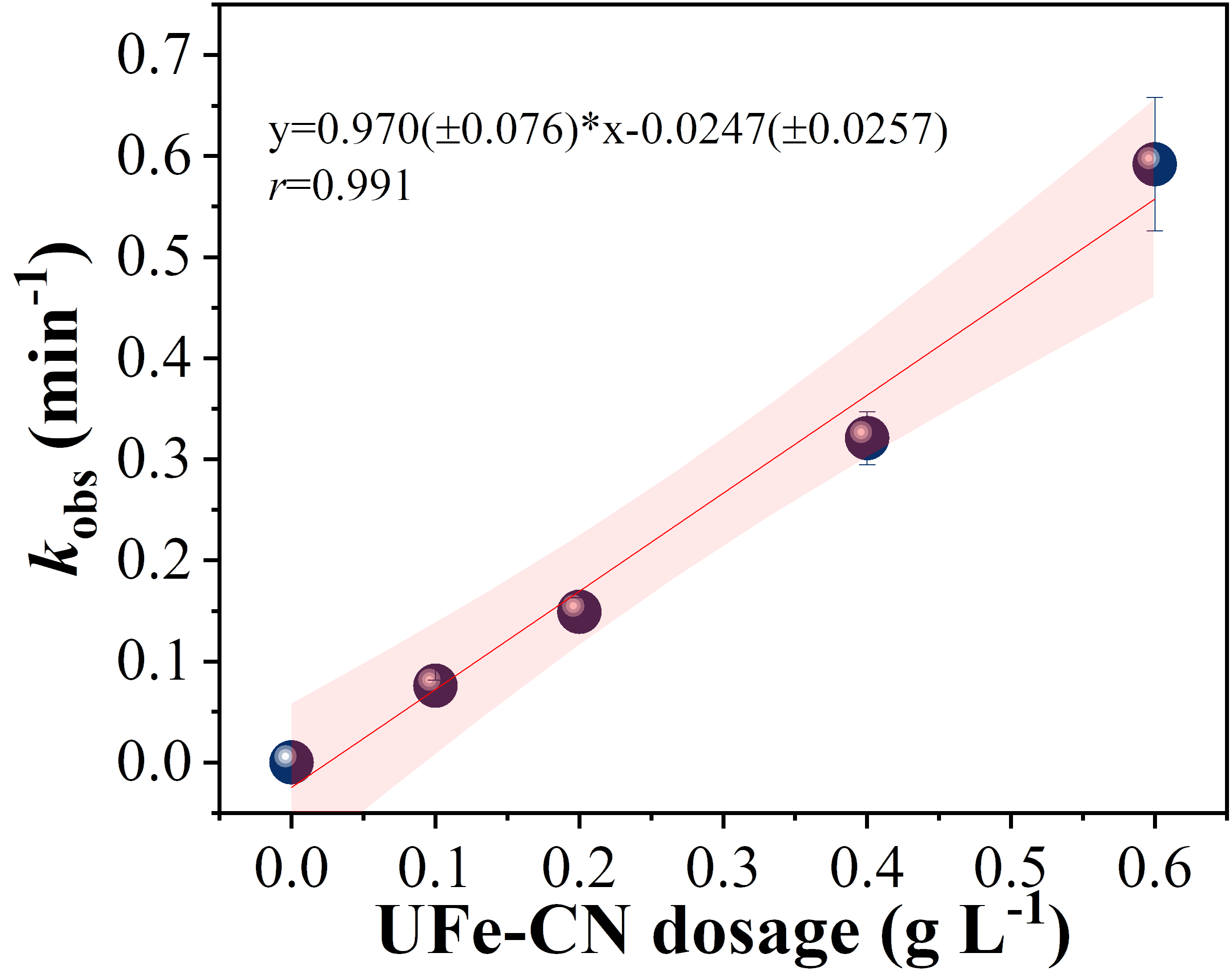


Figure S21. Dependences of *k*_obs_ of 4-NP degradation on the dosages of UFe-CN catalyst. *r*>0.99 indicates a strong linear correlation.


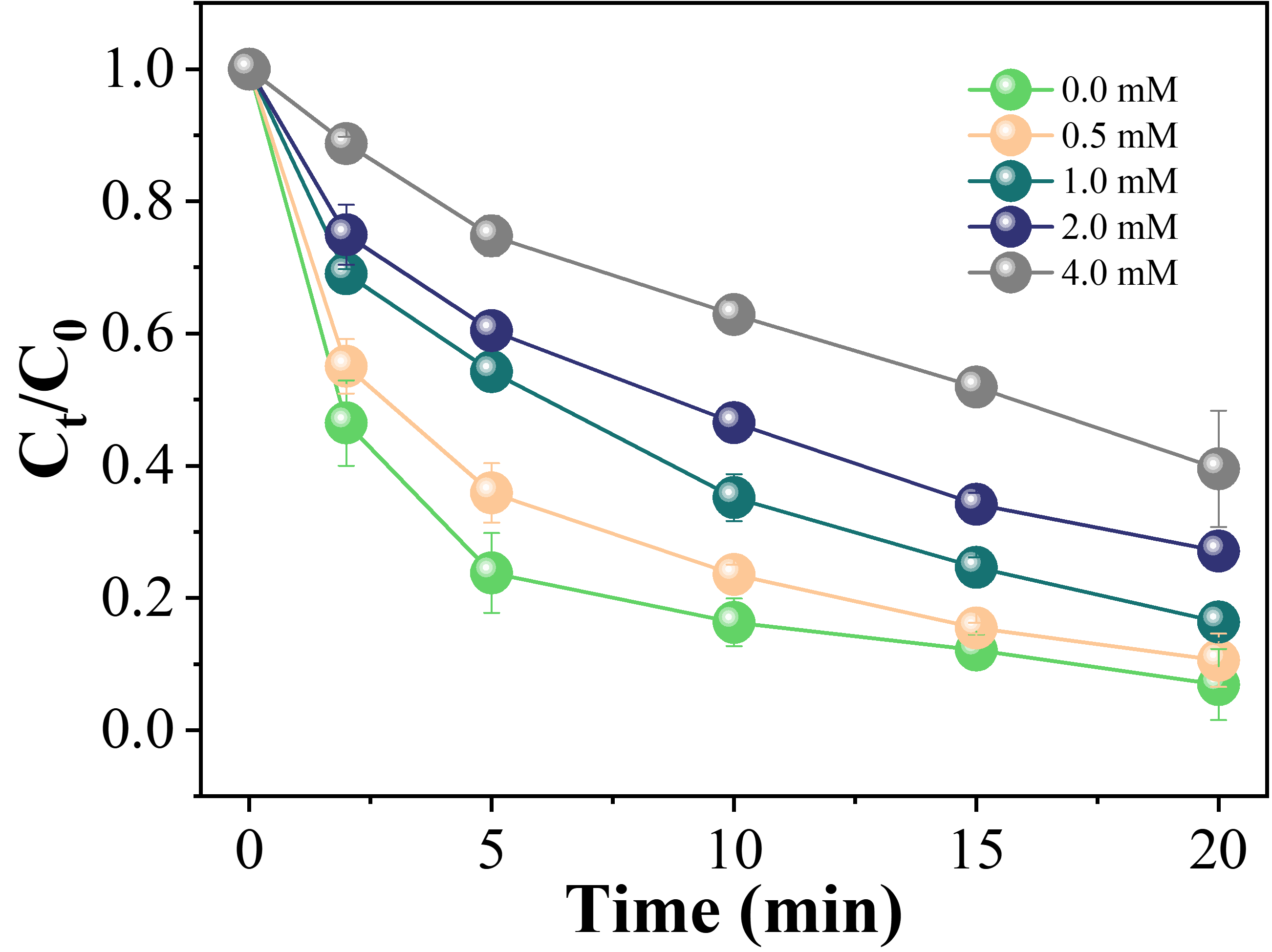


Figure S22. 4-NP degradation in the UFe-CN/PAA Fenton-like system with the addition of various concentrations of carbonate. Experimental conditions: [4-NP] = 20 mg L^-1^, [PAA] = 0.25 mM, [catalyst] = 0.4 g L^-1^, pH_0_ = 7.0, reaction time = 20 min, T = 298.15 K.


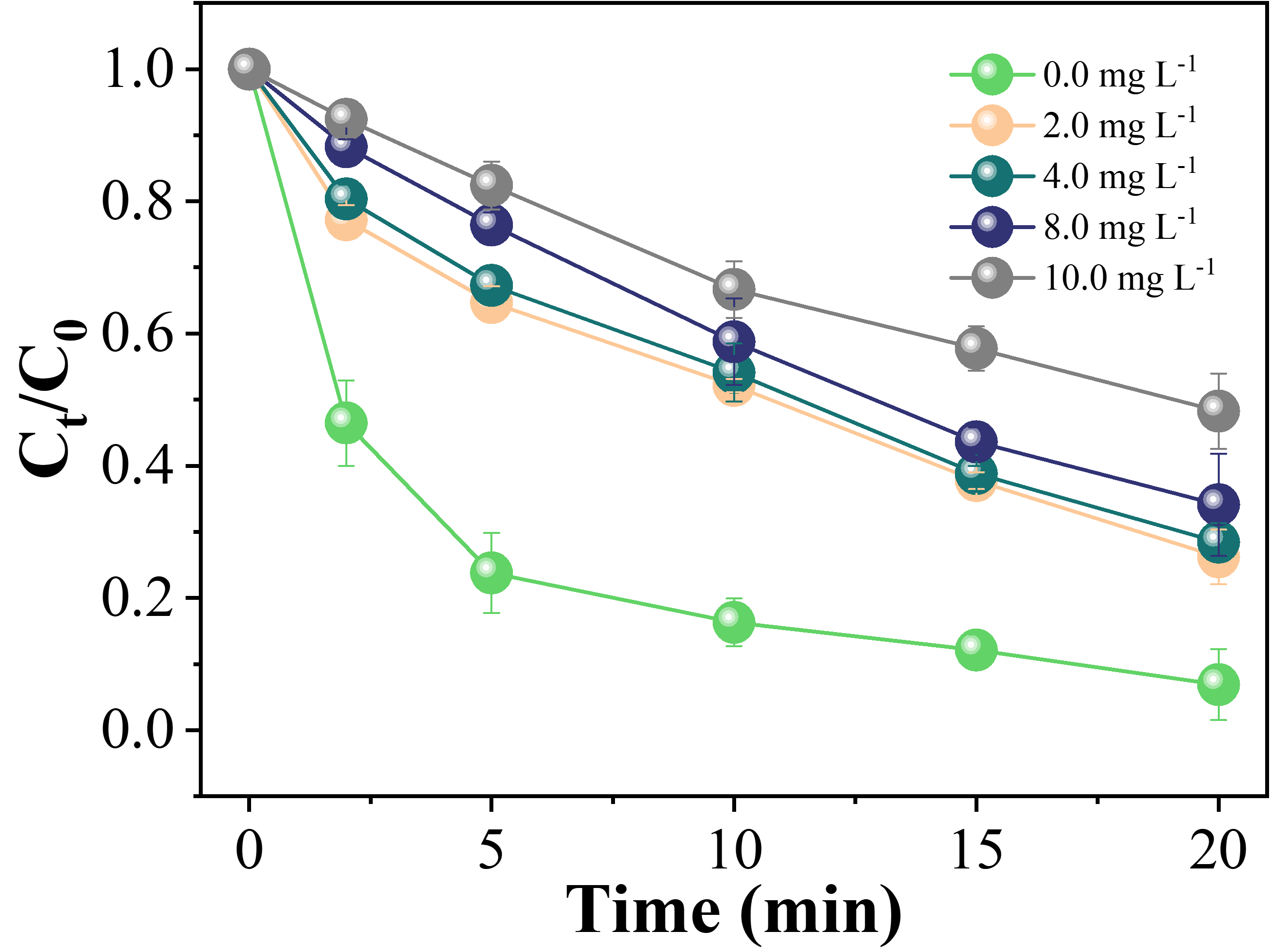


Figure S23. 4-NP degradation in the UFe-CN/PAA Fenton-like system with the addition of various concentrations of HA. Experimental conditions: [4-NP] = 20 mg L^-1^, [PAA] = 0.25 mM, [catalyst] = 0.4 g L^-1^, pH_0_ = 7.0, reaction time = 20 min, T = 298.15 K.


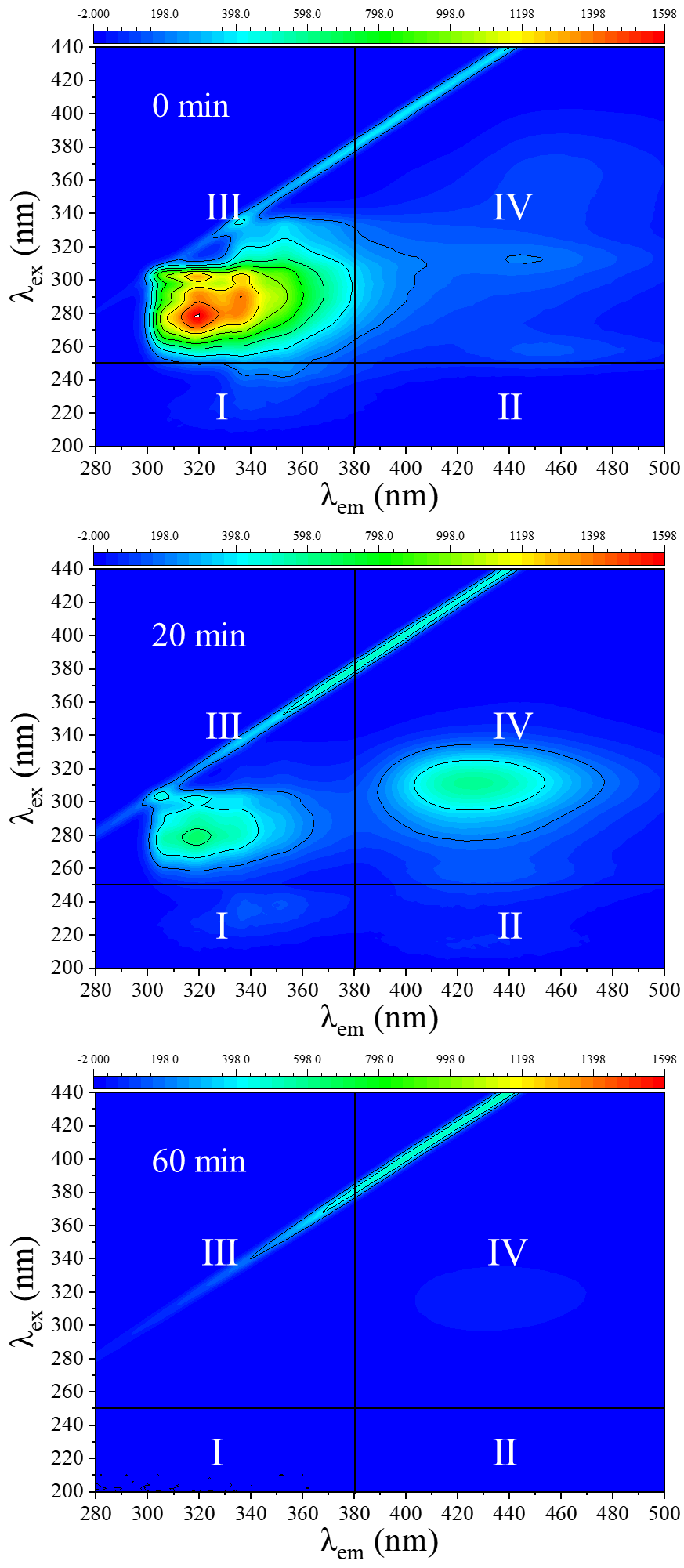


Figure S24. 3D EEM spectra of the another collected TD&F wastewater treated by the UFe-CN/PAA Fenton-like system at reaction times of 0, 20, and 60 min.


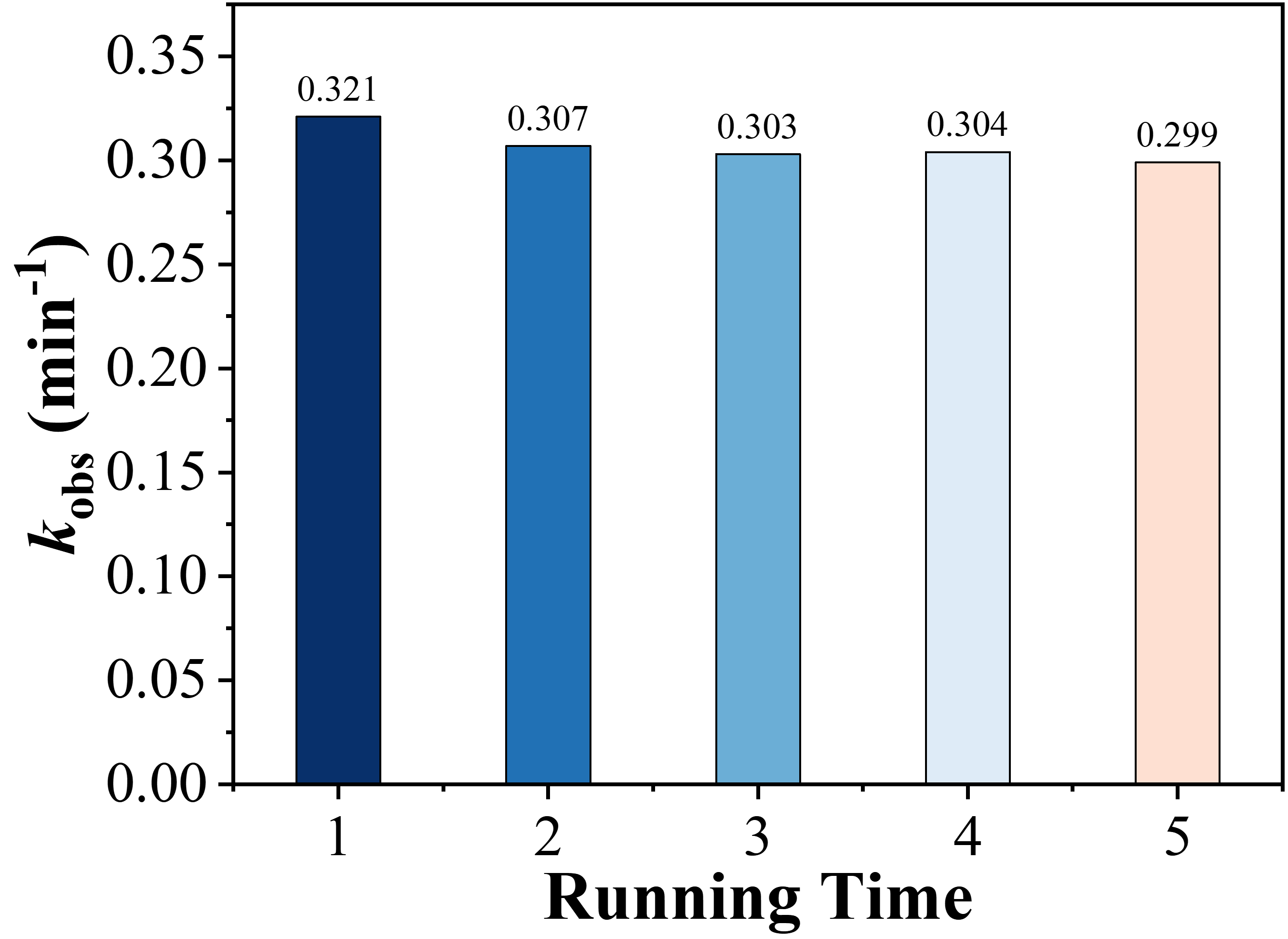


Figure S25. Calculated *k*_obs_ for 4-NP degradation at five consecutive runs by the UFe-CN/PAA Fenton-like system. Each run lasts for 20 min.


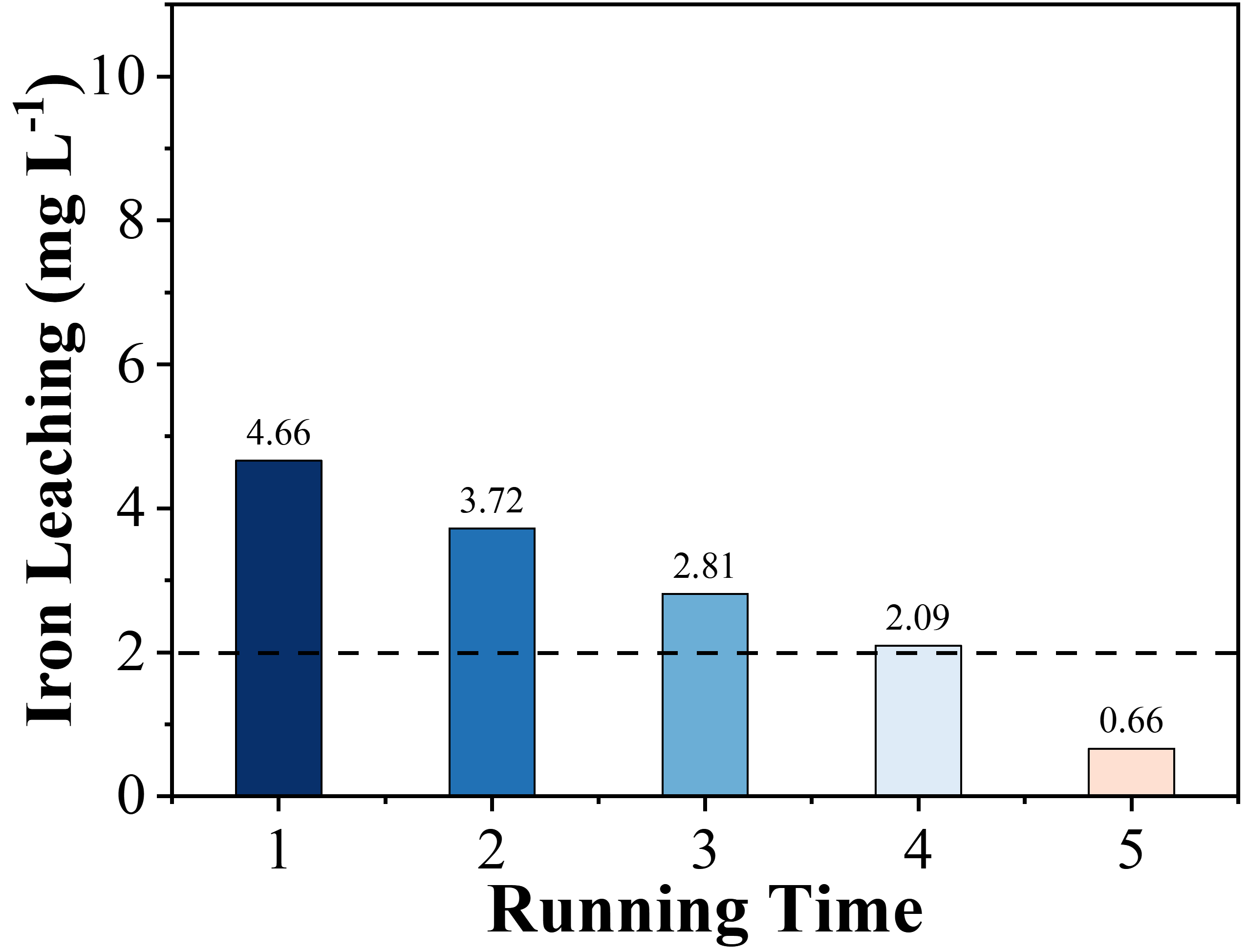


Figure S26. Iron leaching concentrations during five consecutive runs of the UFe-CN/PAA Fenton-like system.


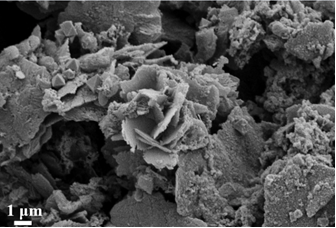


Figure S27. FE-SEM image of the spent UFe-CN catalyst.


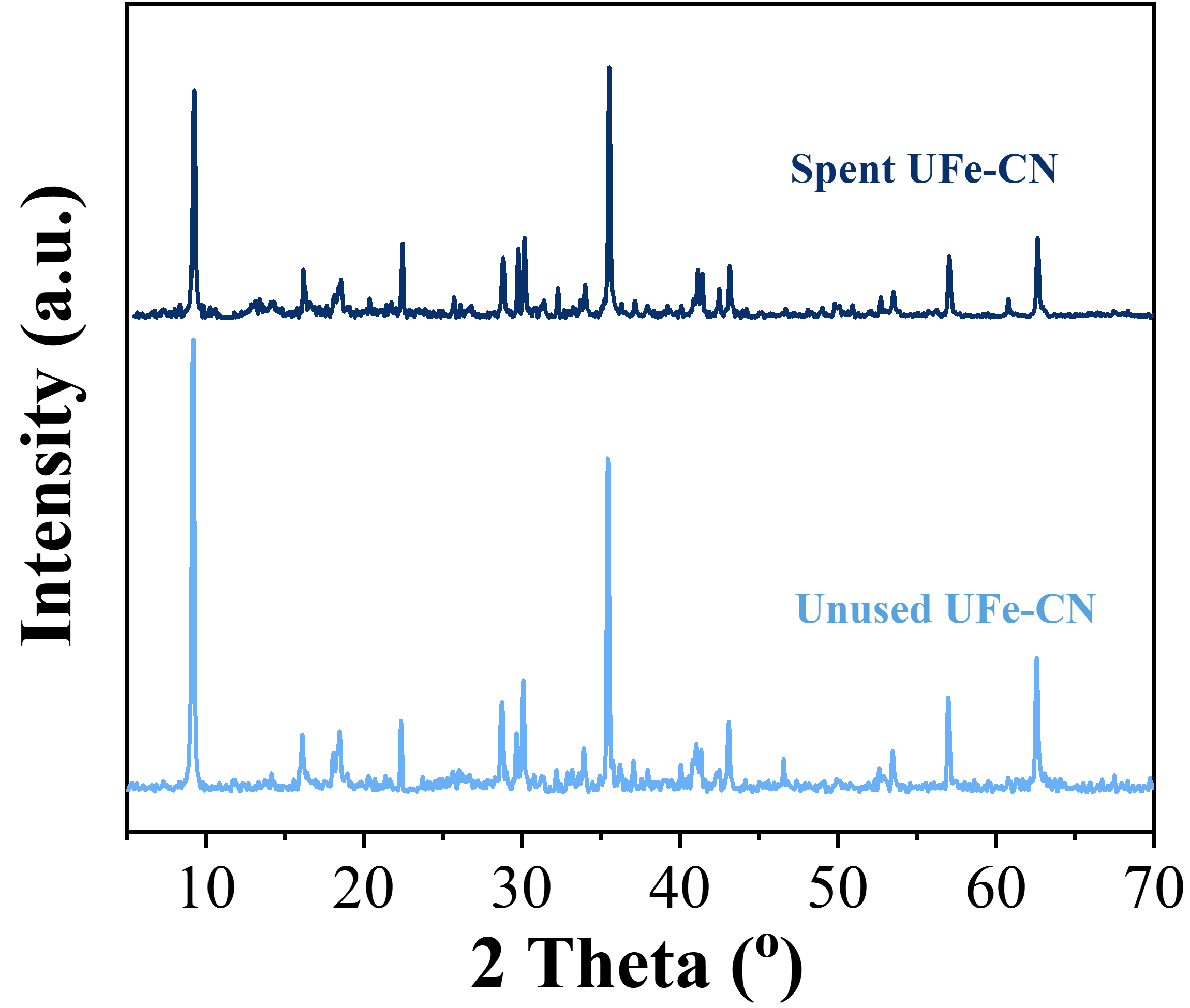


Figure S28. PXRD patterns of the spent and unused UFe-CN catalysts.


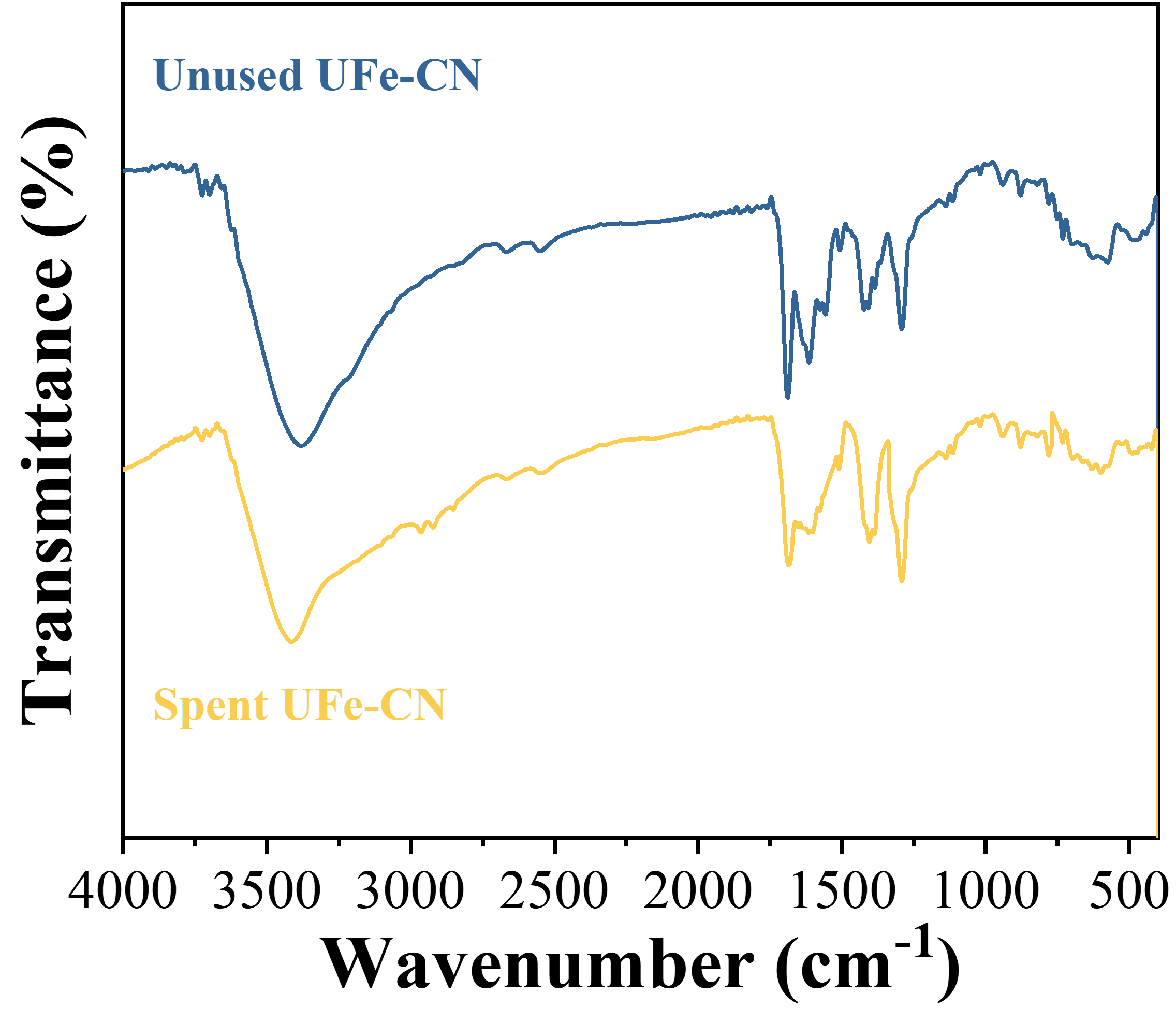


Figure S29. FT-IR spectra of the spent and unused UFe-CN catalysts.

Table S1. Textural properties of UFe-CN and MIL-53(Fe)

| **Sample** | **Specific surface area**  **(m^2^ g^-1^)** | **Total pore volume**  **(cm^3^ g^-1^)** | **Average pore Size**  **(nm)** |
| --- | --- | --- | --- |
| **UFe-CN** | 73.6 | 0.355 | 19.3 |
| **MIL-53(Fe)** | 157.6 | 0.223 | 6.7 |

Table S2. Comparison of 4-NP degradation efficiency between UFe-CN and other catalysts

| **Target pollutants** | **Catalysts** | **Reaction conditions** | **Catalytic performance** | | **Refs.** |
| --- | --- | --- | --- | --- | --- |
|  |  |  | **Removal rate** | ***k*_obs_** |  |
| 4-NP | UFe-CN | pH = 7.0, [catalyst] = 0.4 g L^-1^, [PAA] = 0.25 mM, [4-NP] = 20 mg L^-1^ | 93.1% in 20 min | 0.32 min^-1^ | This work |
| 4-NP | MIL-53(Fe) | pH = 7.0, [catalyst] = 0.4 g L^-1^, [PAA] = 0.25 mM, [4-NP] = 20 mg L^-1^ | 53.0% in 20 min | 0.04 min^-1^ | This work |
| 4-NP | MnO_2_ | pH = 7.0, [catalyst] = 0.4 g L^-1^, [PMS] = 4 g L^-1^, [4-NP] = 25 mg L^-1^ | 97.0% in 80 min | 0.14 min^-1^ | [1] |
| 4-NP | CoFe_2_O_4_@carbon | pH = 6.4, [catalyst] = 0.14 g L^-1^, [PMS] = 0.4 g L^-1^, [4-NP] = 20 mg L^-1^ | 99.0% in 30 min | 0.13 min^-1^ | [2] |
| 4-NP | Co@PCNS | pH = 6.3, [catalyst] = 0.16 g L^-1^, [PMS] = 0.6 g L^-1^, [4-NP] = 20 mg L^-1^ | 99.5% in 12 min | 0.62 min^-1^ | [3] |
| 4-NP | Cu-Co/NC | pH = 5.0, [catalyst] = 0.10 g L^-1^, [PMS] = 1.0 g L^-1^, [4-NP] = 25 mg L^-1^ | >90.0% in 60 min | 0.11 min^-1^ | [4] |
| 4-NP | Cr@CoFe nanoparticles | pH = NA, [catalyst] = 0.50 g L^-1^, [PMS] = 4.4 mM, [4-NP] = 0.21 mM | >80.0% in 40 min | 0.06 min^-1^ | [5] |
| 4-NP | ZIF@GEL | pH = 6.0, [catalyst] = 0.60 g L^-1^, [PMS] = 0.6 g L^-1^, [4-NP] = 25 mg L^-1^ | 90.0% in 60 min | NA | [6] |
| 4-NP | Fe^II^-MIL-53(Fe) | pH = 4.0, [catalyst] = 0.4 g L^-1^, [H_2_O_2_] = 10 mM, [4-NP] = 20 mg L^-1^ | 95.2% in 120 min | 0.02 min^-1^ | [7] |
| 4-NP | MIL-53(Fe) | pH = 4.0, [catalyst] = 0.4 g L^-1^, [H_2_O_2_] = 10 mM, [4-NP] = 20 mg L^-1^ | 65.8% in 120 min | 0.01 min^-1^ | [7] |
| 4-NP | FeVO_4_@CeO_2_ | pH = 7.0, [catalyst] = 0.10 g L^-1^, [H_2_O_2_] = 10 mM, [4-NP] = 20 mg L^-1^, UV irradiation | 95.2% in 60 min | 0.07 min^-1^ | [8] |
| 4-NP | Fe_2_O_3_-Pillared Rectorite | pH = 7.0, [catalyst] = 2.00 g L^-1^, [H_2_O_2_] = 18 mM, [4-NP] = 50 mg L^-1^, Visible light irradiation | 87.0% in 180 min | NA | [9] |
| 4-NP | Fe_3_O_4_ | pH: 7.0; catalyst: 1.5 g L^−1^; [H_2_O_2_]: 620 mM; [4-NP]: 45 mg L^−1^ | > 90% in 10 h | NA | [10] |
| 4-NP | Fe | pH: 3.7; catalyst: 0.5 g L^−1^; [H_2_O_2_]: 2.5 g L^−1^; [4-NP]: 0.5%; Ultrasonic irradiations | 66.4% in 90 min | NA | [11] |

Table S3. Fe(Ⅱ)/Fe(Ⅲ) ratio of UFe-CN catalyst before and after usage

| **Sample** | **Fe(Ⅱ)/Fe(Ⅲ) ratio** |
| --- | --- |
| Unused UFe-CN | 0.52 |
| Spent UFe-CN | 0.51 |

**References**

[1] Z.-G. Zhou, H.-M. Du, Z. Dai, Y. Mu, L.-L. Tong, Q.-J. Xing, S.-S. Liu, Z. Ao, J.-P. Zou, Degradation of organic pollutants by peroxymonosulfate activated by MnO2 with different crystalline structures: Catalytic performances and mechanisms, Chem. Eng. J., 374 (2019) 170-180.

[2] H. Zhu, A. Guo, L. Xian, Y. Wang, Y. Long, G. Fan, Facile fabrication of surface vulcanized Co-Fe spinel oxide nanoparticles toward efficient 4-nitrophenol destruction, J. Hazard. Mater., 430 (2022) 128433.

[3] L. Hu, X. Liu, A. Guo, J. Wu, Y. Wang, Y. Long, G. Fan, Cobalt with porous carbon architecture: Towards of 4-nitrophenol degradation and reduction, Sep. Purif. Technol., 288 (2022) 120595.

[4] X. Li, X. Yan, X. Hu, R. Feng, M. Zhou, L. Wang, Hollow Cu-Co/N-doped carbon spheres derived from ZIFs as an efficient catalyst for peroxymonosulfate activation, Chem. Eng. J., 397 (2020) 125533.

[5] A. Goyal, R. Sharma, S. Bansal, K.B. Tikoo, V. Kumar, S. Singhal, Functionalized core-shell nanostructures with inherent magnetic character: Outperforming candidates for the activation of PMS, Adv. Powder Technol., 29 (2018) 245-256.

[6] W. Ren, J. Gao, C. Lei, Y. Xie, Y. Cai, Q. Ni, J. Yao, Recyclable metal-organic framework/cellulose aerogels for activating peroxymonosulfate to degrade organic pollutants, Chem. Eng. J., 349 (2018) 766-774.

[7] T. Yang, D. Yu, D. Wang, T. Yang, Z. Li, M. Wu, M. Petru, J. Crittenden, Accelerating Fe(Ⅲ)/Fe(Ⅱ) cycle via Fe(Ⅱ) substitution for enhancing Fenton-like performance of Fe-MOFs, "Appl. Catal., B ", 286 (2021) 119859.

[8] G. Eshaq, S. Wang, H. Sun, M. Sillanpaa, Superior performance of FeVO4@CeO2 uniform core-shell nanostructures in heterogeneous Fenton-sonophotocatalytic degradation of 4-nitrophenol, J. Hazard. Mater., 382 (2020) 121059.

[9] G. Zhang, Y. Gao, Y. Zhang, Y. Guo, Fe2O3-Pillared Rectorite as an Efficient and Stable Fenton-Like Heterogeneous Catalyst for Photodegradation of Organic Contaminants, Environ. Sci. Technol., 44 (2010) 6384-6389.

[10] S.-P. Sun, A.T. Lemley, p-Nitrophenol degradation by a heterogeneous Fenton-like reaction on nano-magnetite: Process optimization, kinetics, and degradation pathways, J. Mol. Catal. A: Chem., 349 (2011) 71-79.

[11] Y. Hou, Y. Wang, H. Yuan, H. Chen, G. Chen, J. Shen, L. Li, The enhanced catalytic degradation of SiO2/Fe3O4/C@TiO2 photo-Fenton system on p-nitrophenol, J. Nanopart. Res., 18 (2016) 343.
